# A Ubiquitin-Proteasome Gene Signature for Predicting Prognosis in Patients With Lung Adenocarcinoma

**DOI:** 10.3389/fgene.2022.893511

**Published:** 2022-05-31

**Authors:** Yunliang Tang, Yinhong Guo

**Affiliations:** ^1^ Department of Rehabilitation Medicine, First Affiliated Hospital of Nanchang University, Nanchang, China; ^2^ Department of Oncology, Zhuji People’s Hospital of Zhejiang Province, Zhuji, China

**Keywords:** lung adenocarcinoma, ubiquitin-proteasome, prognosis, predictive modeling, gene signature

## Abstract

**Background:** Dysregulation of the ubiquitin-proteasome system (UPS) can lead to instability in the cell cycle and may act as a crucial factor in both tumorigenesis and tumor progression. However, there is no established prognostic signature based on UPS genes (UPSGs) for lung adenocarcinoma (LUAD) despite their value in other cancers.

**Methods:** We retrospectively evaluated a total of 703 LUAD patients through multivariate Cox and Lasso regression analyses from two datasets, the Cancer Genome Atlas (*n* = 477) and GSE31210 (*n* = 226). An independent dataset (GSE50081) containing 128 LUAD samples were used for validation.

**Results:** An eight-UPSG signature, including *ARIH2*, *FBXO9*, *KRT8*, *MYLIP*, *PSMD2*, *RNF180*, *TRIM28*, and *UBE2V2*, was established. Kaplan-Meier survival analysis and time-receiver operating characteristic curves for the training and validation datasets revealed that this risk signature presented with good performance in predicting overall and relapsed-free survival. Based on the signature and its associated clinical features, a nomogram and corresponding web-based calculator for predicting survival were established. Calibration plot and decision curve analyses showed that this model was clinically useful for both the training and validation datasets. Finally, a web-based calculator (https://ostool.shinyapps.io/lungcancer) was built to facilitate convenient clinical application of the signature.

**Conclusion:** An UPSG based model was developed and validated in this study, which may be useful as a novel prognostic predictor for LUAD.

## Introduction

Non-small cell lung cancer (NSCLC) constitutes approximately 40%–50% of total lung cancers, which are the leading cause of tumor-associated mortality worldwide (Wu et al., 2019; Siegel et al., 2020). NSCLC is still difficult to diagnose in the early stages due to its insidious progression, and more than two thirds of patients are only diagnosed in its advanced stages. Despite the existence of various chemo/physical/immunological therapies, the 5-year survival rate for patients with any type of lung cancer remains very low (Bade and Dela Cruz, 2020). Histologically, lung adenocarcinoma (LUAD) is the most common form of NSCLC, and comprises nearly 40% of all cases (Hua et al., 2020). Therefore, it is necessary to explore the specific molecular pathogenesis of LUAD and develop novel therapeutic targets for its treatment (Wang J. et al., 2020; Tang et al., 2020).

The ubiquitin-proteasome system (UPS) is a major intracellular protein degradation pathway in eukaryotic organisms, which controls a wide range of physiological processes and disease conditions, such as transcription, translation, DNA repair, and the cell cycle (Chen and Chen, 2016). The UPS primarily comprises the E1, E2, and E3 ubiquitin ligases, the proteasome, and various deubiquitinating enzymes. Dysregulation of the UPS can contribute to the inhibition of cell cycle regulation and disorder of cancer cell metabolism, ultimately leading to carcinogenesis (Dang et al., 2021). In fact, the UPS is already an important target in various cancer treatments, including the use of proteasome and ubiquitin E3 ligases as therapeutic targets for various chemotherapies (Manasanch and Orlowski, 2017). Abnormal expression of different UPS gene families alters proteolysis, inhibiting the proliferation and metastasis of LUAD (He et al., 2021). Thus, evaluating the relationship between UPS and tumorigenesis might provide novel insights into LUAD pathology and facilitate better patient prognosis. However, the prognostic values of UPS-related genes (UPSGs) and their molecular function in LUAD remain poorly understood.

Here, we investigated changes in UPSGs expression in the data downloaded from The Cancer Genome Atlas (TCGA) and Gene Expression Omnibus (GEO) databases and then built a scoring model by classifying LUAD patients based on a multi-UPSGs signature, in combination with other clinicopathological factors, to improve our ability to predict the prognosis of LUAD patients, thereby helping to guide clinical treatment. This model might be meaningful for the development of comprehensive therapeutic approaches for LUAD patients.

## Methods

### Data Collection

The transcriptomic (HTSeq-FPKM), demographic, and clinical information of patients with LUAD was collected from TCGA (training set) and GEO databases (validation set) for evaluations. First, TCGA-LUAD dataset was curated to remove any cases with incomplete survival data or a follow-up time of less than <30 days, leaving a total of 477 patients with LUAD who were then included in this study. GSE50081 were applied as validation datasets (Okayama et al., 2012; Der et al., 2014), where GSE31210 comprised a total of 226 primary LUAD patients at stage I–II, with a median age of 61 years and an age range of 30–76 years; the cohort included 105 male and 121 female patients. GSE50081 comprised a total of 128 LUAD cases with a median age of 70.38 years and an age range of 40.16–85.91 years; this cohort consisted of 98 male and 83 female patients. The clinical characteristics of all three datasets analyzed in this study are summarized in Table 1.

**TABLE 1:** Clinical information analyzed in this study.

Features	TCGA-LUAD	GSE31210	GSE50081
Sample	477	226	128
Mean age (years; range)	66 (33–88)	61 (30–76)	70.38 (40.16–85.91)
Gender			
Male	215	105	65
Female	254	121	63
Stage			
I	253	168	92
II	113	58	36
III	78	-	-
IV	25	-	-
Status			
Alive	314	191	76
Dead	115	35	52
Platform	Illumina HiSeqV2	HG-U133_Plus_2	HG-U133_Plus_2

The mutation profiles for each of the UPSGs identified in LUAD patients were downloaded from the cBioPortal database (https://www.cbioportal.org/) (Gao et al., 2013). A total of 804 UPSGs were identified in a previous study and used as the basis of our evaluations in this study (Supplementary Table S1) (Wang et al., 2021).

### Establishing a Prognostic UPSG Signature

To narrow the number of candidate UPSGs, we conducted univariate Cox regression analysis on TCGA-LUAD and GSE31210 datasets. The overlapping overall survival (OS)-related genes were selected for further study. These targets were then cross-validated using least absolute shrinkage and selection operator (LASSO) regression, which then produced a list of potential predictors with nonzero coefficients using the R packages “glmnet” and “survival.” Finally, we performed multivariate Cox regression analysis on TCGA-LUAD dataset to confirm the identity of highly correlated genes and construct the OS gene signature using the following risk score model:

$$\text{Risk score=}\sum_{\text{i}=0}^{N}\left(\beta_{\text{i}}\times Exp_{i}\right),$$

where N represents the number of UPSGs included in the signature, Expi represents the mRNA level of these genes, and βi represents the regression coefficient obtained using the Cox regression analyses.

The patients in each dataset were classified as high- or low-risk, using the median risk score as the cutoff value, and the receiver operating characteristic (ROC) curves were created using the “survivalROC” package in R. The AUC values were calculated to assess the predictive potential of the UPSG signature.

### Validation of the Prognostic Signature

The prognostic UPSG model was then applied to the GSE50081 validation dataset. Validation dataset was split into high- and low-risk groups for evaluation using Kaplan-Meier curve analysis, time-dependent ROC analysis, and patient outcome distribution to assess the categorization of the UPSG signature.

### Subgroup Analyses of the UPSG Signature

The clinical usefulness of the prognostic UPSG signature was evaluated through stratification analysis that was performed to investigate the differences in the prognosis of LUAD patients presenting with different clinical characteristics. Based on both characteristics and risk score, the LUAD cases were divided into low- and high-risk groups, and Cox regression was applied to analyze these subgroups.

### Estimation of Immune Cell Infiltration

To determine whether and how UPSGs affected the tumor immune microenvironment, Cell type Identification by Estimating Relative Subsets of RNA Transcripts (CIBERSORT) was used to predict the fractions of immune cell types between the high- and low-risk cohorts. Eventually, 22 types of differentially expressed immune cells associated with specific clinicopathologic characteristics in the high- and low-risk cohorts were identified and depicted as a landscape map.

### Construction and Evaluation of the Nomogram

The UPSG signature and clinicopathological predictors were combined to construct a nomogram for TCGA training set. A calibration curve was prepared to assess the agreement between the nomogram model and actual observation. The time-ROC curves and calibration plots were generated, and decision curve analyses (DCA) was used to assess the predictive accuracy of the signature.

## Results

### Establishing a UPSG Signature

We first defined OS-related UPSGs through a univariate Cox regression analysis from TCGA-LUAD and GSE31210 datasets. The two datasets comprised 95 and 157 OS-related UPSGs, respectively. Then, we reduced the number of OS-related UPSGs by taking the intersection of TCGA-LUAD and GSE31210 datasets. Eventually, 33 overlapping OS-related UPSGs were identified (Supplementary Figure S1). Subsequently, the 33 UPSGs were used in a Lasso-Cox proportional hazards regression and ten-fold cross-validation analyses designed to construct the best gene signature; eventually, 13 UPSGs were identified for downstream analysis (Supplementary Figure S1). Furthermore, a multivariate Cox regression was used to evaluate the Lasso results (Supplementary Figure S1). The prognostic risk score according to the expression of the eight UPSGs was determined as follows: Risk score = ARIH2 × (−0.075232) + FBXO9 × (−0.055219) + KRT8 × 0.00059 + MYLIP × (−0.034945) + PSMD2 × 0.006608 + RNF180 × (−0.123541) + TRIM28 × 0.004102 + UBE2V2 × 0.031981 (Table 2).

**TABLE 2:** The prognostic ubiquitin-proteasome system-related genes identified by using LASSO COX regression.

Symbol	Coefficient	Name
ARIH2	−0.075232	Ariadne RBR E3 ubiquitin protein ligase 2
FBXO9	−0.055219	F-box protein 9
KRT8	0.000590	Keratin 8
MYLIP	−0.034945	Myosin regulatory light chain interacting protein
PSMD2	0.006608	Proteasome 26S subunit, non-ATPase 2
RNF180	−0.123541	Ring finger protein 180
TRIM28	0.004102	Tripartite motif containing 28
UBE2V2	0.031981	Ubiquitin conjugating enzyme E2 V2

Moreover, mutation analysis of the eight UPSGs included in the prognostic signature revealed that *PSMD2*, *RNF180*, *UBE2V2*, *KRT8*, and *MYLIP* were the most frequently mutated genes. Notably, amplification was the most common type of mutation, and *UBE2V2*, *PSMD2*, and *FBXO9* were frequently amplified in LUAD (Supplementary Figure S1).

### Evaluating the Prognostic Significance of the UPSG Signature

**FIGURE 1:**
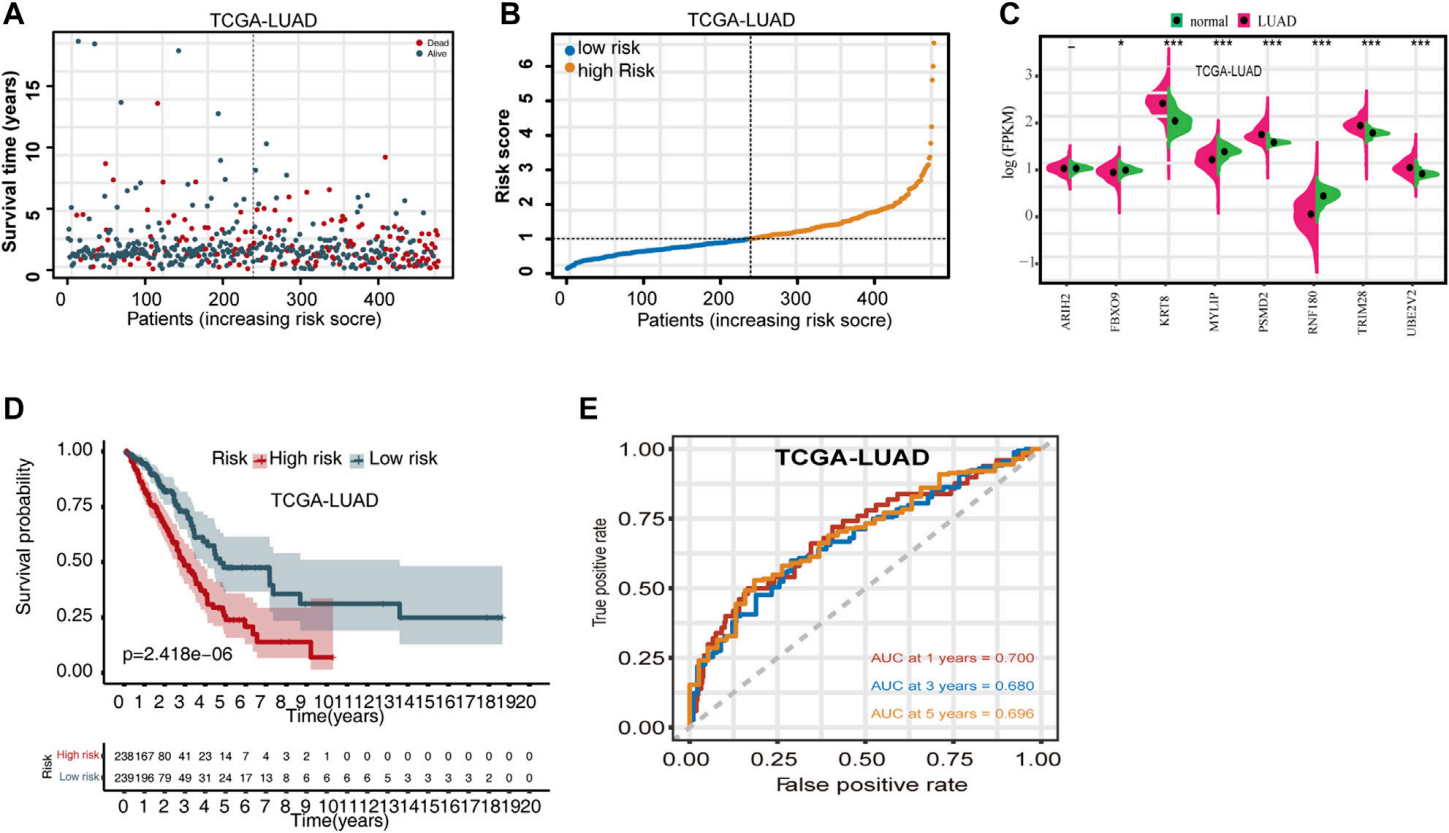
Evaluating the predictive power of the UPSG signature in TCGA-LUAD training set. **(A)** Distribution of overall survival status ranked by risk score. **(B)** Risk score distribution across OS prediction. **(C)** Comparison of the eight-UPSG mRNA expression levels between normal and tumor tissues. **(D)** Kaplan-Meier survival analysis of patients in the high- and low-risk cohorts. **(E)** Time-dependent receiver operating characteristic curves of the signature for predicting 1-, 3-, and 5-year overall survival rates. *p < 0.05, **p < 0.01, and ***p < 0.001 represent difference between normal and tumor tissues.

Using the risk score calculation formula derived from the multivariate Cox regression, the risk score for each TCGA-LUAD patient was calculated. Based on the median risk score, patients were separated into high- and low-risk cohorts. The distribution of OS status ranked by risk score is presented in Figure 1A, and the risk score distribution for OS prediction in TCGA dataset is shown in Figure 1B. Differences in expression of the eight UPSGs between normal and tumor tissues is shown in Figure 1C. The results show that *KRT8*, *PSMD2*, *TRIM28*, and *UBE2V2* are significantly overexpressed in tumor tissues, while the expression level of *FBXO9, MYLIP*, and *RNF180* is significantly reduced in LUAD (*p* < 0.05).

We then evaluated the differences in the survival rates of the high- and low-risk groups. The results revealed that the high-risk group exhibited a significantly poorer OS rate than the low-risk group (Figure 1D). In addition, we then used time-dependent ROC curves and their AUC values to evaluate the prognostic significance of this stratification. AUC values were estimated to be 0.700, 0.680, and 0.696 for 1-, 3-, and 5-year OS, respectively (Figure 1E). Moreover, subset analyses using stratification *via* clinicopathologic feature revealed that this UPSG signature exhibited significant predictive value in most subgroups (Table 3).

**TABLE 3:** Stratified survival analyses and clinical characteristics with UPSGs prognostic signature in the TCGA-LUAD cohort (*n* = 477).

Characteristics	No.		%	Overall survival	
	High-risk	High-risk		HR (95% CI)	*p* Value
Age (years)					
<65	109	103	44.4	2.058 (1.235–3.429)	0.006
≥65	129	136	55.6	2.241 (1.448–3.469)	0.000
Sex					
Male	116	104	46.1	2.828 (1.711–4.675)	0.000
Female	122	135	53.9	1.686 (1.076–2.644)	0.023
Stage					
I	106	147	53.0	1.695 (0.973–2.950)	0.062
II	68	45	23.7	1.594 (0.847–2.998)	0.148
III	46	32	16.4	2.385 (1.218–4.673)	0.011
IV	16	9	5.2	2.683 (0.744–9.673)	0.132
Unknow	2	6	1.7	--	-
T stage					
T1	65	94	33.3	1.578 (0.826–3.016)	0.167
T2	137	117	53.2	2.085 (1.345–3.230)	0.001
T3	27	16	9.0	5.539 (1.257–24.408)	0.024
T4	8	10	3.8	2.823 (0.690–11.549)	0.149
Unknow	1	2	0.6	-	-
M stage					
M0	156	157	65.6	2.181 (1.447–3.286)	0.000
M1	16	8	5.0	3.726 (0.821–16.902)	0.088
Unknow	66	74	29.4	-	-
N stage					
N0	133	174	64.4	1.984 (1.228–3.205)	0.005
N1	59	31	18.9	1.556 (0.816–2.965)	0.179
N2	40	27	14.0	2.133 (1.044–4.358)	0.038
N3	1	1	0.4	-	-
Unknow	5	6	2.3	-	-

**FIGURE 2:**
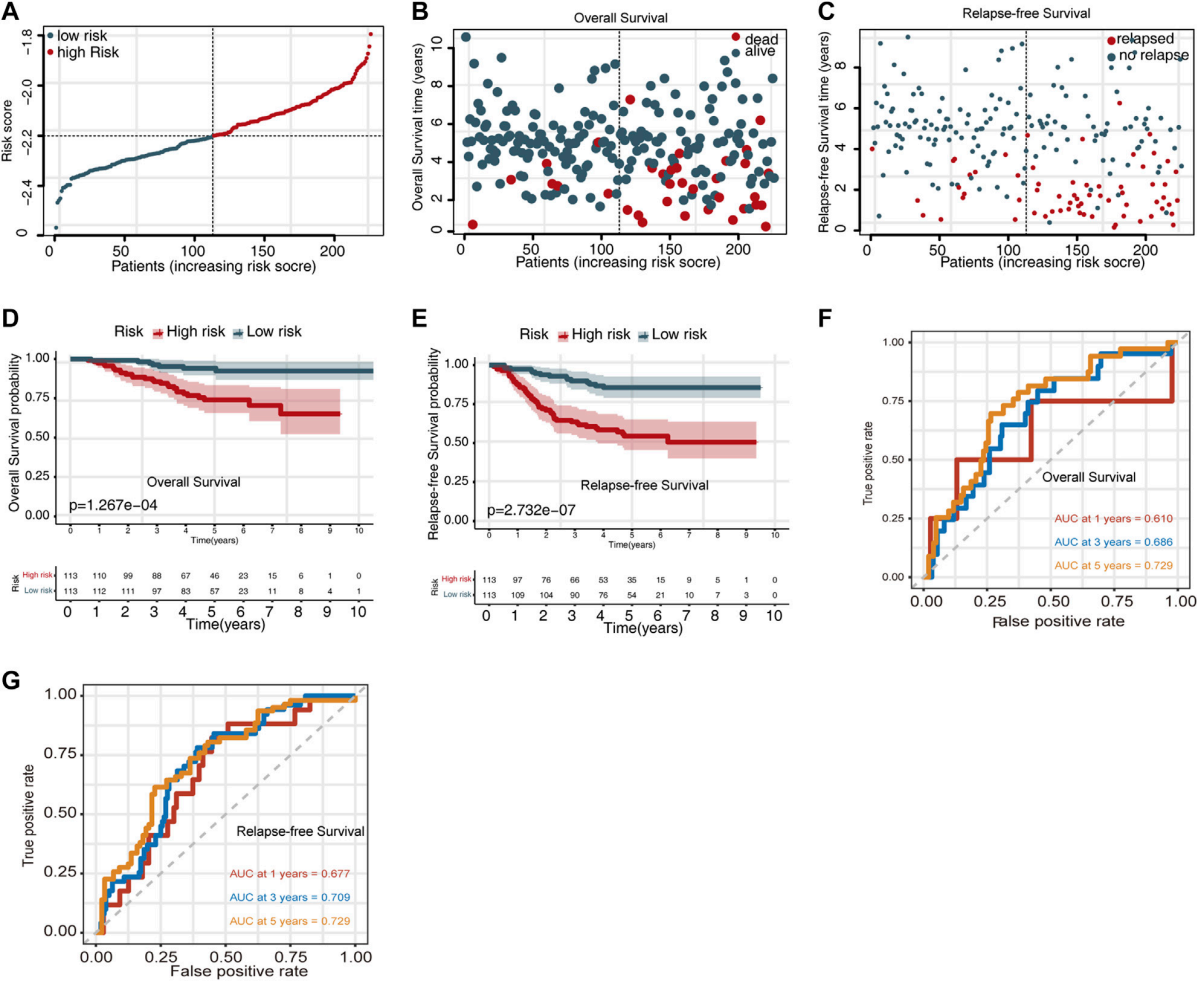
Evaluating the predictive power of the UPSG signature in GSE31210 dataset. **(A)** Risk score distribution for survival prediction. **(B)** Distribution of the overall survival status ranked by risk score. **(C)** Distribution of the relapse-free survival status ranked by risk score. **(D)** Overall survival analysis of patients in the high- and low-risk groups. **(E)** Relapse-free survival analysis of patients in the high- and low-risk groups. **(F)** Time-dependent receiver operating characteristic curves of the signature for predicting 1-, 3-, and 5-year overall survival. **(G)** Time-dependent receiver operating characteristic curves of the signature for predicting 1-, 3-, and 5-year relapse-free survival.

Moreover, we also evaluated the prognostic significance of the UPSG signature in the GSE31210 dataset. The distribution of the risk scores for the GSE31210 dataset is presented in Figure 2A. The risk score distribution for OS prediction is shown in Figure 2B, and the risk score distribution for relapsed-free survival (RFS) prediction is shown in Figure 2C. Survival analysis indicated that patients in the high-risk group had worse OS and RFS compared with the low-risk group (Figures 2D,E). Using the time-dependent ROC curves, the AUC values for 1-, 3-, and 5-year OS prediction were estimated to be 0.610, 0.686, and 0.729, respectively (Figure 2F), while those for RFS prediction were 0.677, 0.709, and 0.729, respectively (Figure 2G).

### Validating the Prognostic Value of the UPSG Signature

To validate the prognostic value of the UPSG signature, we verified its prediction performance in the GSE50081 datasets.

**FIGURE 3:**
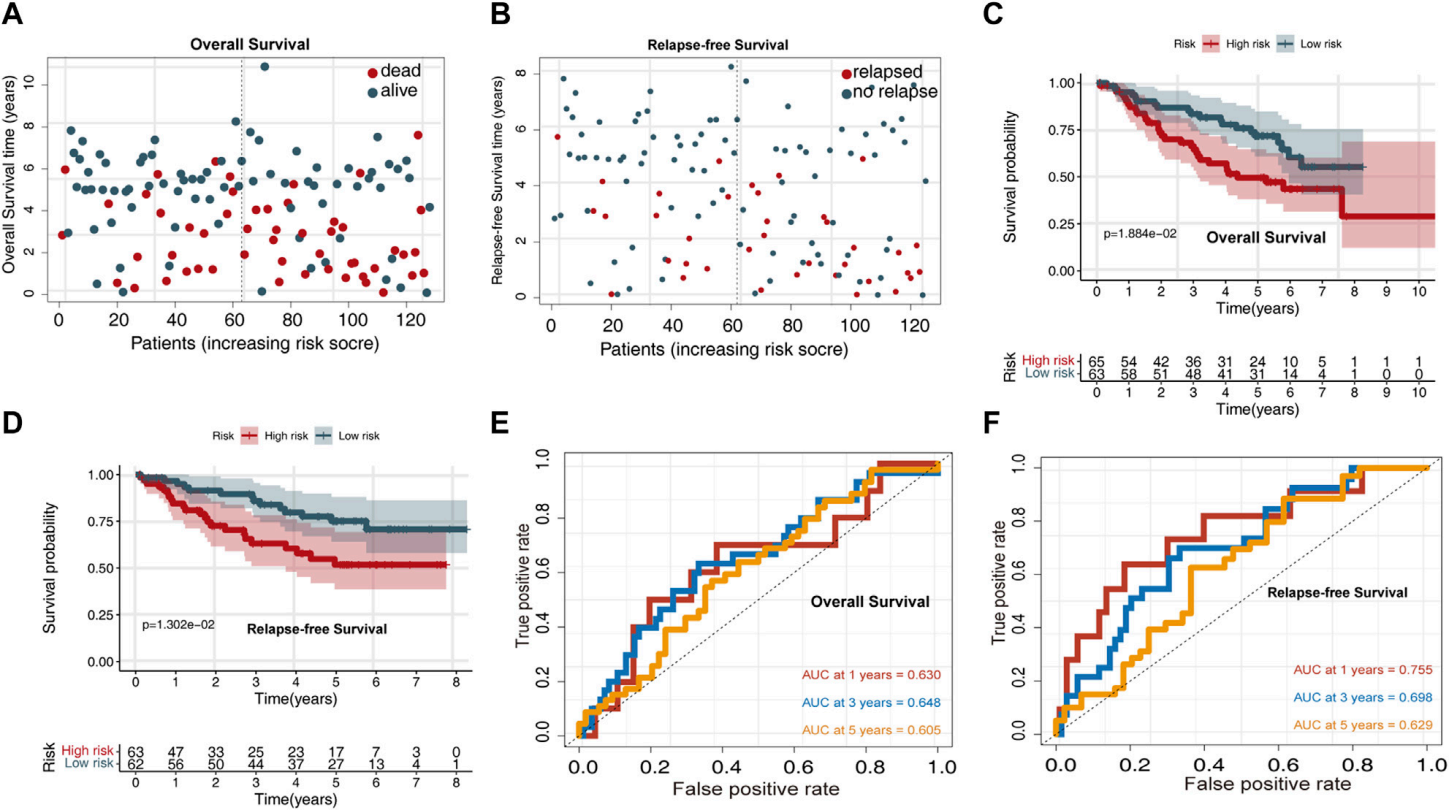
Validating the predictive power of the UPSG signature in the GSE50081 validation set. **(A)** Distribution of overall survival status ranked by risk score. **(B)** Distribution of relapse-free survival status ranked by risk score. **(C)** Overall survival analysis of patients in the high- and low-risk groups. **(D)** Relapse-free survival analysis of patients in the high- and low-risk groups. **(E)** Time-dependent receiver operating characteristic curves of the signature for predicting 1-, 3-, and 5-year overall survival. **(F)** Time-dependent receiver operating characteristic curves of the signature for predicting 1-, 3-, and 5-year relapse-free survival.

**FIGURE 4:**
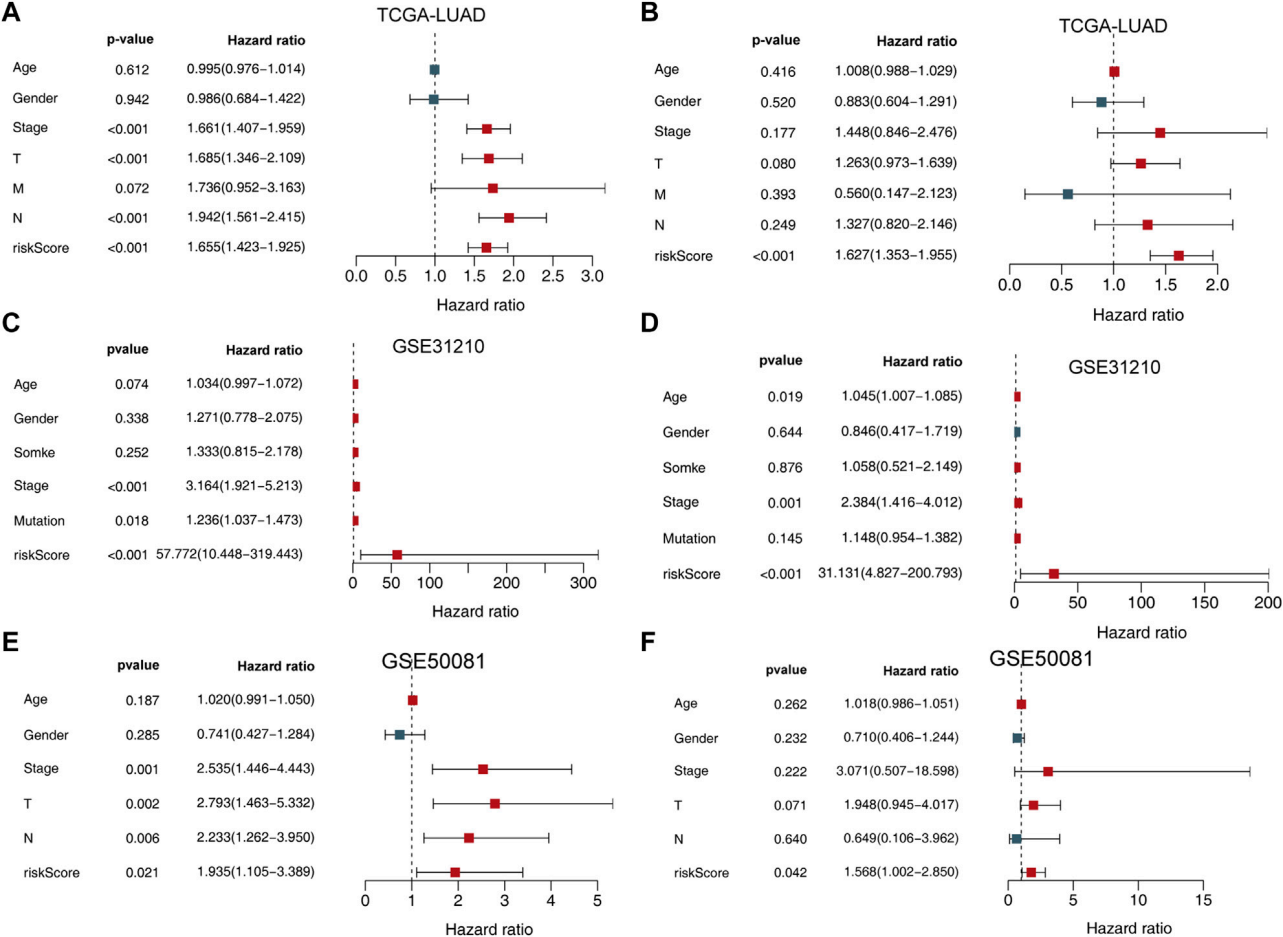
Univariate and multivariate analysis of the influence of specific clinical characteristics in the outcome of LUAD patients. **(A)** Univariate analysis for TCGA-LUAD training set. **(B)** multivariate analysis of TCGA-LUAD training set. **(C)** Univariate analysis for the GSE31210 dataset. **(D)** Multivariate analysis for the GSE31210 dataset. **(E)** Univariate analysis for the GSE50081 validation set. **(F)** Multivariate analysis for the GSE50081 validation set.

The risk score distribution for OS prediction in the GSE50081 dataset is displayed in Figure 3A, and the risk score distribution for RFS prediction is shown in Figure 4B. Survival analysis indicated that the high-risk group exhibited a worse OS and RFS than the low-risk group (Figures 3C,D). Time-dependent ROC curves for OS prediction revealed AUC values for 1-, 3-, and 5-year OS of 0.63, 0.648, and 0.605, respectively (Figure 3E). Time-dependent ROC curves for RFS prediction revealed AUC values for 1-, 3-, and 5-year RFS of 0.755, 0.698, and 0.629, respectively (Figure 3F).

We then used both univariate and multivariate Cox regression analyses to determine whether this signature and other clinicopathological features were independent predictors of LUAD survival. The results indicated that the UPSG signature may serve as an independent prognostic indicator in both TCGA-LUAD (Figures 4A,B), GSE31210 (Figures 4C,D) datasets, and GSE50081 (Figures 4E,F) dataset.

### Correlation Between UPSGs and Clinicopathological Factors

**FIGURE 5:**
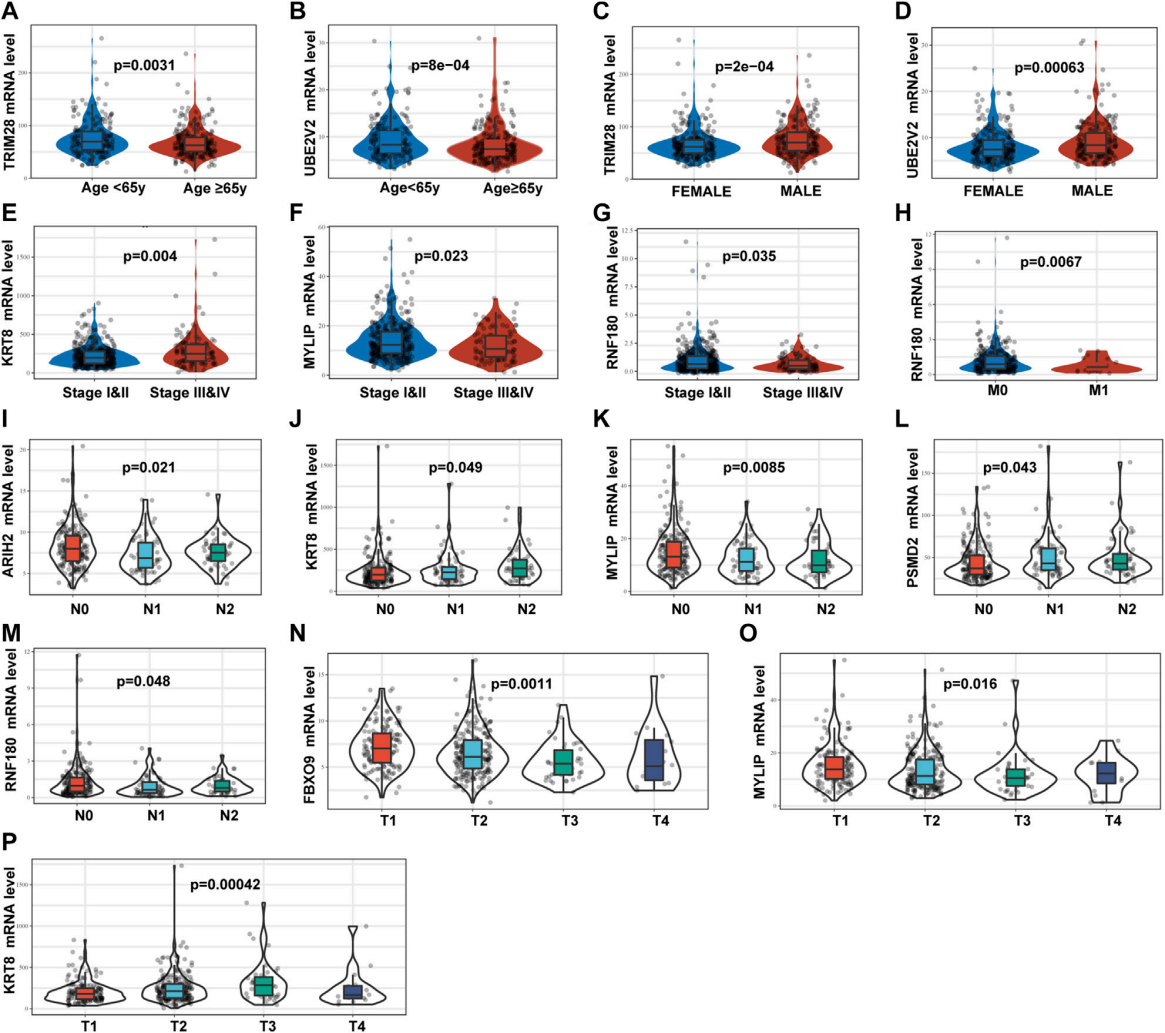
Relationships between the mRNA expression of identified UPSGs and clinicopathological factors in patients with LUAD based on the TCGA-LUAD cohort. **(A)** TRIM28 expression and age. **(B)** UBE2V2 expression and age. **(C)** TRIM28 expression and gender. **(D)** UBE2V2 expression and gender. **(E)** KRT8 expression and tumor stage. **(F)** MYLIP expression and tumor stage. **(G)**RNF180 expression and tumor stage. **(H)**RNF180 expression and M stage. **(I)** ARIH2 expression and N stage. **(J)** KRT8 expression and N stage. **(K)** MYLIP expression and N stage. **(L)** PSMD2 expression and N stage. **(M)** RNF180 expression and N stage. **(N)** FBXO9 expression and T stage. **(O)** MYLIP expression and T stage. **(P)** KRT8 expression and T stage.

We analyzed the relevance of the signature genes in relation to various clinicopathological parameters of LUAD using the data from TCGA-LUAD training set. These analyses revealed that *TRIM28*, *UBE2V2*, *KRT8*, *MYLIP*, *FBXO9*, *ARIH2*, and *RNF180* were all significantly correlated with specific clinicopathological features (Figures 5A–P).

### Evaluating the Infiltrating Immune Cell Ratios in High- and Low-Risk LUAD Patients

**FIGURE 6:**
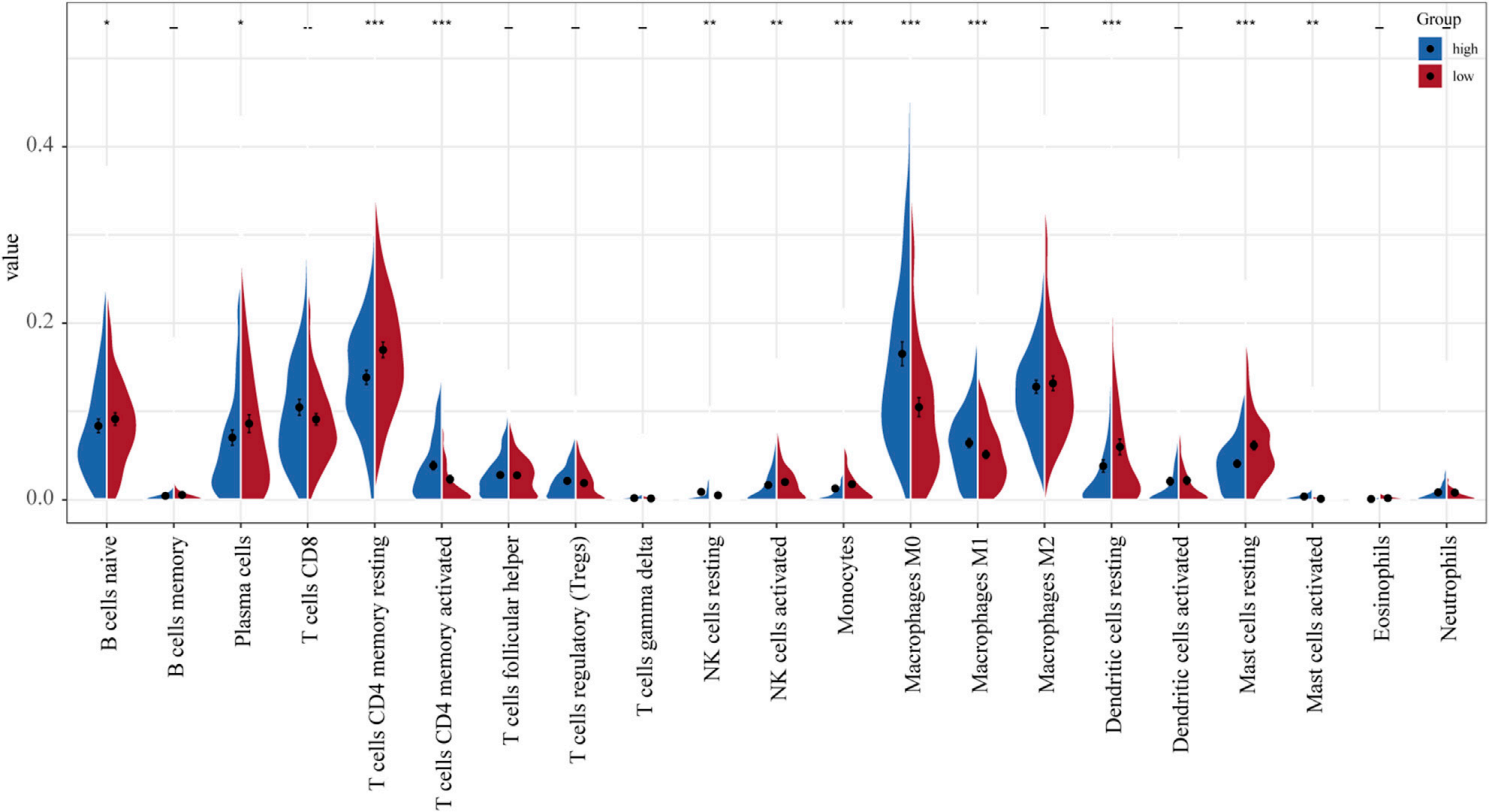
Differences in the distribution of immune cell infiltration between normal and tumor tissues (relative proportion of immune cells in each group).

TCGA-LUAD gene expression data were uploaded to the CIBERSORT platform to evaluate any changes in the overall proportions of the 22 immune cell types in response to their allocation into high- and low-risk groups. Compared to low-risk patients, high-risk patients exhibited an increased proportion of activated CD4 memory T cells, resting NK cells, M0 macrophages, M1 macrophages, and activated mast cells and a reduced proportion of naïve B cells, plasma cells, resting CD4 memory T cells, activated NK cells, resting dendritic cells, and resting mast cells (Figure 6).

### Establishing and Evaluating a Novel LUAD Nomogram

**FIGURE 7:**
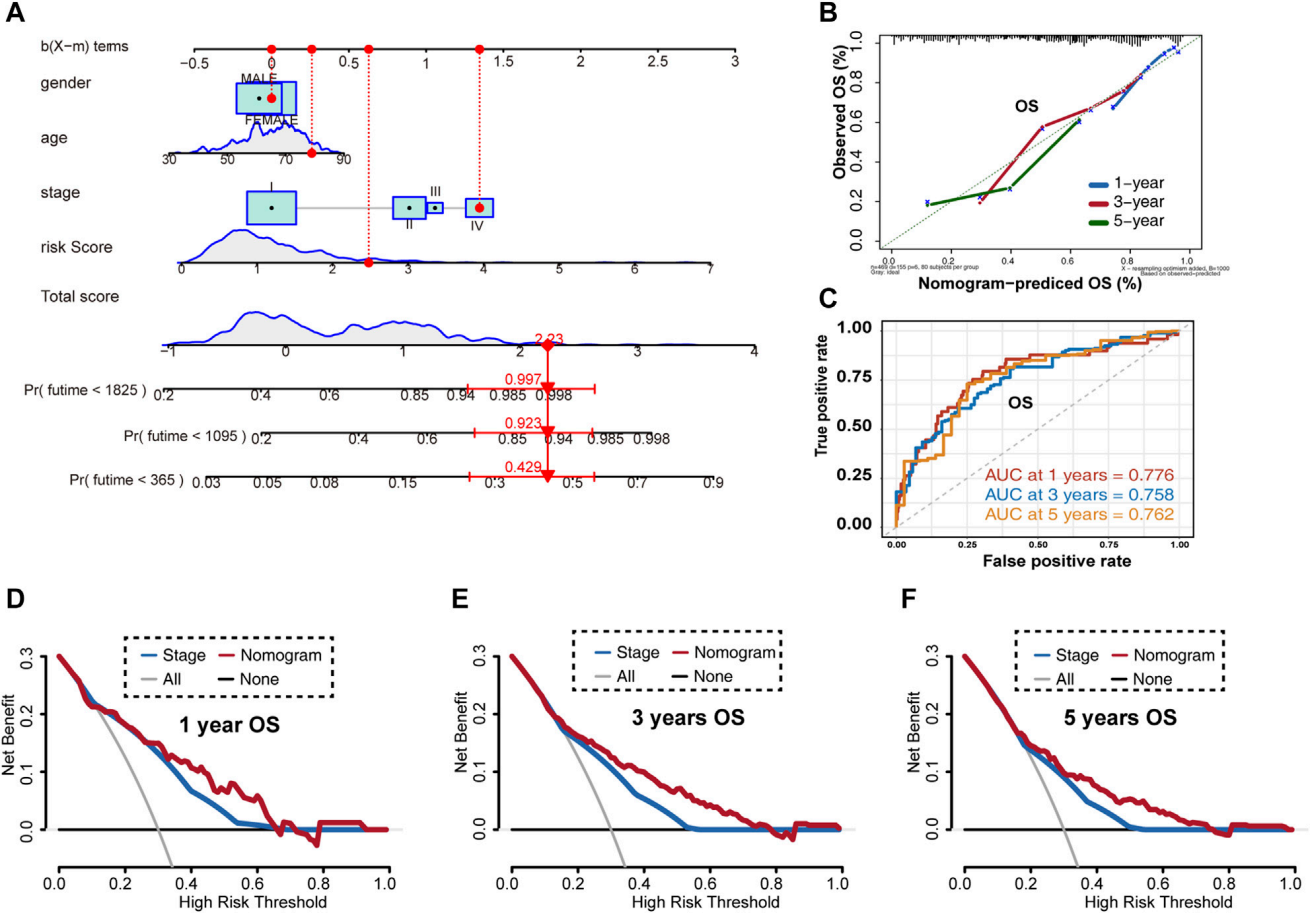
Overall survival prediction using the LUAD nomogram based on TCGA-LUAD training set. **(A)** Nomogram predictions for 1-, 3-, and 5-year overall survival in LUAD patients. **(B)** Calibration plot of the predictive model. **(C)** Time-dependent receiver operating characteristic curves for nomogram-mediated prediction of 1-, 3-, and 5-year overall survival in LUAD patients. **(D)** Decision curve analysis for 1-year overall survival rates. **(E)** Decision curve analysis for 3-year overall survival rates. **(F)** Decision curve analysis for 5-year overall survival rates. OS, overall survival.

Given these results, we established a novel nomogram for accurate clinical prediction of LUAD outcomes, using age, gender, stage, and signature risk score in TCGA-LUAD (Figure 7A), and then evaluated its precision using a calibration plot to predict the 1-, 3-, and 5-year OS rates for these samples, all of which exhibited strong correlation with the nomogram findings (Figure 7B). The AUC values for the nomogram at 1-, 3-, and 5-year OS were 0.776, 0.758, and 0.762, respectively (Figure 7C). Furthermore, results of DCA for predicting 1-, 3-, and 5-year survival probability confirmed our findings. As shown in Figures 7D–F, the nomogram presented higher net benefit than stage in predicting OS.

**FIGURE 8:**
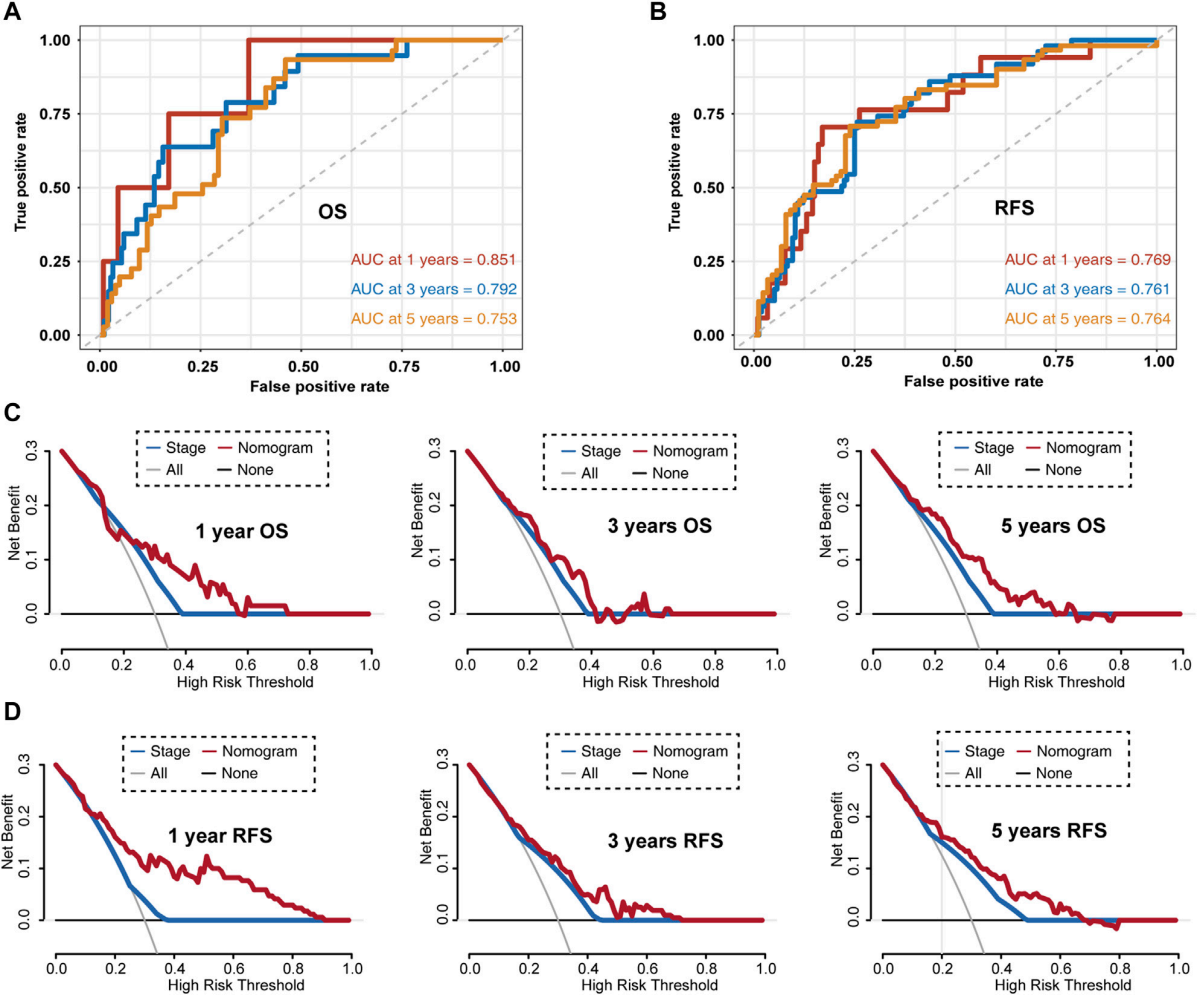
Evaluating LUAD survival prediction nomogram based on the GSE31210 dataset. **(A)** Time-dependent receiver operating characteristic curves for nomogram prediction of 1-, 3-, and 5-year overall survival rates. **(B)** Time-dependent receiver operating characteristic curves for nomogram mediated prediction of 1-, 3-, and 5-year relapse-free survival in LUAD patients. **(C)** Decision curve analysis for overall survival. **(D)** Decision curve analysis for relapse-free survival. OS, overall survival; RFS, relapsed-free survival.

Then, we evaluated this nomogram in the GSE31210 dataset. The AUC values of the nomogram at 1-, 3-, and 5-year OS in the ROC curves were 0.851, 0.792, and 0.735, respectively (Figure 8A). The AUC values of the nomogram at 1-, 3-, and 5-year RFS in the ROC curves were 0.769, 0.761, and 0.764, respectively (Figure 8B). Furthermore, DCA for predicting 1-, 3-, and 5-year OS and RFS probability confirmed these results. As shown in Figures 8C,D.

Moreover, we also evaluated this nomogram in the GSE50081 dataset. The AUC values of the nomogram at 1-, 3-, and 5-year OS in the ROC curves were 0.652, 0.693, and 0.676, respectively (Supplementary Figure S1A). The AUC values of the nomogram at 1-, 3-, and 5-year RFS in the ROC curves were 0.728, 0.688, and 0.703, respectively (Supplementary Figure S1B). Furthermore, DCA for predicting 1-, 3-, and 5-year OS and RFS probability confirmed these results. As shown in Supplementary Figures S1C,D the nomogram based on the best model exhibited higher net benefit than stage during OS and RFS prediction.

**FIGURE 9:**
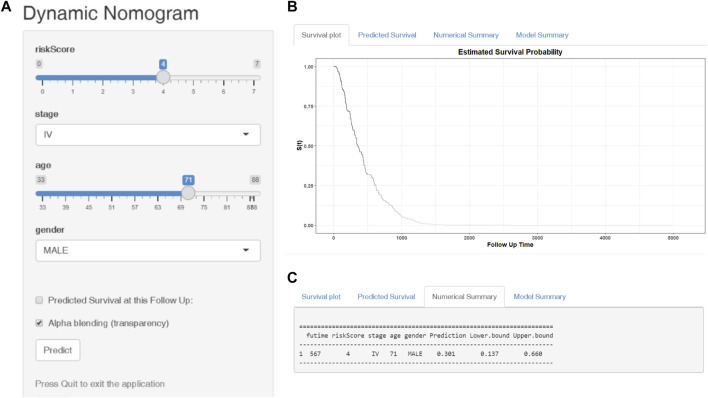
Web-based dynamic nomogram for predicting the overall survival of LUAD patients (https://ostool.shinyapps.io/lungcancer/). **(A)** Input interface: You can enter the related variables of participants in this interface. **(B)** Survival plot: It represents participants’ survival probability with different follow up time. **(C)** Numerical summary: It shows the actual values of probability and 95% confidence intervals.

Finally, we established a web-based tool (https://ostool.shinyapps.io/lungcancer/) for estimating the OS of patients with LUAD (Figures 9A,B) for clinical application of our nomogram and gene signature.

For convenient clinical use and visualization of the prognostic model, we developed a simple-to-operate web-based model (https://ostool.shinyapps.io/lungcancer/) to predict the prognosis of LUAD based on the established nomogram (Figures 9A,B). Estimated LUAD survival probabilities can be obtained by drawing a perpendicular line from the total point axis to the outcome axis.

## Discussion

Our understanding of the ubiquitin-proteasome system has opened up a new era of anticancer treatment through the maintenance and protection of protein quality control and homeostasis (Li X. et al., 2018; Narayanan et al., 2020). Various UPS inhibitors are being evaluated in clinical trials as novel cancer therapeutics (Johnson, 2015). However, the prognostic value of UPSGs in LUAD patients remains poorly understood. Therefore, complete comprehensive analysis of UPSGs is urgently required to explore their clinical significance in LUAD.

Here, we used RNA-seq data from both TCGA and GEO databases to establish a UPSG prognostic risk signature incorporating *ARIH2*, *FBXO9*, *KRT8*, *MYLIP*, *PSMD2*, *RNF180*, *TRIM28*, and *UBE2V2* for LUAD patients. Kaplan-Meier survival and ROC curve analyses based on both the training and validation sets indicated that this risk signature exhibited excellent fitting and predictive capacity. Thus, these results could help in the development of novel prognostic biomarkers and provide clinical research ideas for LUAD.

Among the eight UPSGs included in the signature, several genes have previously been explored in terms of their association with LUAD. ARIH2 (Ariadne RBR E3 ubiquitin ligase 2), an E3 ligase whose expression levels are regulated by PABPN1, is one of the most important factors involved in regulating biological function (Raz et al., 2014). A previous study has reported that loss of *ARIH2* significantly accelerates the development of resistant HCC827 cells, but the specific mechanism of action needs further investigation (Zeng et al., 2019). FBXO9 (F-box only protein), an SCF (Skp1-Cul-F-box)-type ubiquitin ligase for Neurog2 (Liu J. A. et al., 2020), has been studied in various malignancies. Liu et al. reported that inhibition of FBXO9 increased proteasome activity and sensitivity to bortezomib, suggesting that FBXO9 has an important role in bortezomib susceptibility (Hynes-Smith et al., 2019). However, FBXO9 does not have E3 ligase activity by itself and it is a substrate recognition subunit of an E3 ligase complex. KRT8 (keratin 8), a type II basic intermediate filament protein, is essential for the development and metastasis of various cancers, including LUAD (Xie et al., 2019). Wang et al. explored the expression of KRT8 in LUAD and found that it was overexpressed in tumor tissues and associated with poorer prognosis (Wang W. et al., 2020); these results are consistent with those of our study. MYLIP (myosin regulatory light chain interacting protein), an E3 ubiquitin ligase, which is associated with the regulation of cell motility and migration (Chen et al., 2017), has been found to play a critical role in malignant tumors (Zhao et al., 2020). Ni et al. reported that MYLIP regulates the growth and metastasis-related phenotypes of cervical cancer cells Ni et al. (2021), while the knockdown of *PSMD2* (proteasome 26S subunits, non-ATPase 2) has been reported to suppress tumor cell proliferation (Matsuyama et al., 2011; Li Y. et al., 2018; Okumura et al., 2018). PSMD2 has also been shown to be associated with the acquisition of metastatic phenotypes and poor prognosis in various lung cancers (Matsuyama et al., 2011). RNF180 (ring finger protein 180), an important member of the E3 ubiquitin ligase family, act as a tumor suppressor by inhibiting the proliferation, invasion, and migration of gastric cancer cells (Wu et al., 2020). Previous studies have also indicated that reduced RNF180 expression levels were associated with poor biological behavior in A549 and HCC827 cells (Liu H. et al., 2020). TRIM28 (tripartite motif-containing protein 28) is involved in several cellular processes including gene transcription, cell growth and differentiation, genome stability, immunity, development, and carcinogenesis (Hatakeyama, 2011; Iyengar and Farnham, 2011; Messerschmidt et al., 2012; Sio et al., 2012). Chen et al. reported that TRIM28 is a tumor suppressor in the early transformation process of lung cancer but acts as an oncogene in advanced stages of this disease Chen et al. (2014). UBE2V2 (ubiquitin-conjugating enzyme E2 variant 2) has sequence similarity to other ubiquitin-conjugating enzymes but lacks the conserved cysteine residues that are essential for the catalytic activity of E2 (Dikshit et al., 2018; Zhao et al., 2018). Hua et al. reported that UBE2V2 was identified as an independent prognostic indicator for LUAD and might function as a therapeutic target Hua et al. (2021).

The UPS is also regarded as an important player in the regulation of immune cell function and immune response (Çetin et al., 2021). Here, we evaluated the differences in the proportions of 22 immune cell types between low- and high-risk LUAD groups. Our results showed that compared to low-risk patients, high-risk patients have a higher proportion of activated CD4 memory T cells, resting NK cells, M0 macrophages, M1 macrophages, and activated mast cells and a reduced proportion of naïve B cells, plasma cells, resting CD4 memory T cells, activated NK cells, resting dendritic cells, and resting mast cells. These data indicate that this novel gene signature represents differences in immune infiltration, suggesting that immune status may have a critical effect on prognosis.

Given these promising results, we constructed a more reliable and individualized clinical prediction model, using our eight-UPSG signature and several other clinical features to establish a nomogram. The AUC values of the nomogram for TCGA-LUAD data at 1-, 3-, and 5-year OS were 0.776, 0.758, and 0.762, respectively, while those for the GSE31210 dataset were 0.851, 0.792, and 0.735, respectively (Figure 8A). In addition, the AUC values of the nomogram at 1-, 3-, and 5-year RFS in this dataset were 0.769, 0.761, and 0.764, respectively (Figure 8B). DCA results also suggested that the nomogram based on the best model exhibited a larger net prognostic benefit than stage. For the convenience of clinical application, we established a web-based tool for estimating the OS of patients with LUAD. Taken together, these results may enable us to deliver higher accuracy in prognosis prediction for patients with LUAD.

To the best of our knowledge, this is the first study to establish a UPSG signature for predicting the survival of patients with LUAD. However, this study had some limitations. First, we did not investigate the detailed mechanisms of each of the eight UPSGs identified in this study; these underlying mechanisms warrant further investigation through both *in vitro* and *in vivo* studies. Second, the inclusion of clinical indicators in the model seems to be insufficient; other factors such as, radiotherapy, surgery type, and targeted drug use might affect the prognosis of LUAD. Third, only two GEO datasets were applied for validation; however, further validation using larger datasets is required.

Compared to a signature developed on a complete set of genes, our UPSG signature has a major advantage. We made a comprehensive exploration of the prognostic role of UPSGs in LUAD from a bioinformatic perspective. Compared to the large and complex genome signature, our risk had a better clinical operability which was calculated by several simple UPSGs. However, our research also has a major disadvantage. As our research was based on UPSGs, other prognosis genes were ignored and not included in this study.

In summary, we established a novel signature model for LUAD prognosis based on an eight-UPSG signature, which accurately predicted prognosis of LUAD patients. This signature may serve as an effective tool for designing personalized therapies and guiding medical decisions for LUAD patients in the future. Moreover, these UPSGs in the signature are promising targets for pharmacological and gene therapy in the future.

## Data Availability

The original contributions presented in the study are included in the article/Supplementary Material, further inquiries can be directed to the corresponding author.

## Abbreviations

LUAD, lung adenocarcinoma; TCGA, The Cancer Genome Atlas; GEO, Gene Expression Omnibus; OS, overall survival; RFS, relapsed-free survival; UPS, ubiquitin-proteasome system; UPSG, ubiquitin-proteasome system related gene; AUC, area under the curve; ROC, receiver operating characteristic; DCA, decision curve analysis.
