# Supplementary material for: A Ubiquitin-Proteasome Gene Signature for Predicting Prognosis in Patients With Lung Adenocarcinoma
Source: Front Genet. 2022 May 31;13:893511. doi: 10.3389/fgene.2022.893511 (PMC9194557; doi:10.3389/fgene.2022.893511)
Supplement: Supplementary file 1 [file Table1.DOCX]

| **Table S1. ubiquitin proteasome system genes** | | | |
| --- | --- | --- | --- |
| **Gene ID** | **Gene Symbol** | **Name** | **Species** |
| E1s |  |  |  |
| 7317 | UBA1 | ubiquitin like modifier activating enzyme 1(UBA1) | Homo sapiens |
| 10054 | UBA2 | ubiquitin like modifier activating enzyme 2(UBA2) | Homo sapiens |
| 9039 | UBA3 | ubiquitin like modifier activating enzyme 3(UBA3) | Homo sapiens |
| 79876 | UBA5 | ubiquitin like modifier activating enzyme 5(UBA5) | Homo sapiens |
| 55236 | UBA6 | ubiquitin like modifier activating enzyme 6(UBA6) | Homo sapiens |
| 7318 | UBA7 | ubiquitin like modifier activating enzyme 7(UBA7) | Homo sapiens |
| 10533 | ATG7 | autophagy related 7(ATG7) | Homo sapiens |
| 27304 | MOCS3 | molybdenum cofactor synthesis 3(MOCS3) | Homo sapiens |
| 8883 | NAE1 | NEDD8 activating enzyme E1 subunit 1(NAE1) | Homo sapiens |
| 10055 | SAE1 | SUMO1 activating enzyme subunit 1(SAE1) | Homo sapiens |
| E2s |  |  |  |
| 7319 | UBE2A | ubiquitin conjugating enzyme E2 A(UBE2A) | Homo sapiens |
| 7320 | UBE2B | ubiquitin conjugating enzyme E2 B(UBE2B) | Homo sapiens |
| 11065 | UBE2C | ubiquitin conjugating enzyme E2 C(UBE2C) | Homo sapiens |
| 7321 | UBE2D1 | ubiquitin conjugating enzyme E2 D1(UBE2D1) | Homo sapiens |
| 7322 | UBE2D2 | ubiquitin conjugating enzyme E2 D2(UBE2D2) | Homo sapiens |
| 7323 | UBE2D3 | ubiquitin conjugating enzyme E2 D3(UBE2D3) | Homo sapiens |
| 51619 | UBE2D4 | ubiquitin conjugating enzyme E2 D4 (putative)(UBE2D4) | Homo sapiens |
| 7324 | UBE2E1 | ubiquitin conjugating enzyme E2 E1(UBE2E1) | Homo sapiens |
| 7325 | UBE2E2 | ubiquitin conjugating enzyme E2 E2(UBE2E2) | Homo sapiens |
| 10477 | UBE2E3 | ubiquitin conjugating enzyme E2 E3(UBE2E3) | Homo sapiens |
| 140739 | UBE2F | ubiquitin conjugating enzyme E2 F (putative)(UBE2F) | Homo sapiens |
| 7326 | UBE2G1 | ubiquitin conjugating enzyme E2 G1(UBE2G1) | Homo sapiens |
| 7327 | UBE2G2 | ubiquitin conjugating enzyme E2 G2(UBE2G2) | Homo sapiens |
| 7328 | UBE2H | ubiquitin conjugating enzyme E2 H(UBE2H) | Homo sapiens |
| 7329 | UBE2I | ubiquitin conjugating enzyme E2 I(UBE2I) | Homo sapiens |
| 51465 | UBE2J1 | ubiquitin conjugating enzyme E2 J1(UBE2J1) | Homo sapiens |
| 118424 | UBE2J2 | ubiquitin conjugating enzyme E2 J2(UBE2J2) | Homo sapiens |
| 3093 | UBE2K | ubiquitin conjugating enzyme E2 K(UBE2K) | Homo sapiens |
| 7332 | UBE2L3 | ubiquitin conjugating enzyme E2 L3(UBE2L3) | Homo sapiens |
| 9246 | UBE2L6 | ubiquitin conjugating enzyme E2 L6(UBE2L6) | Homo sapiens |
| 9040 | UBE2M | ubiquitin conjugating enzyme E2 M(UBE2M) | Homo sapiens |
| 7334 | UBE2N | ubiquitin conjugating enzyme E2 N(UBE2N) | Homo sapiens |
| 389898 | UBE2NL | ubiquitin conjugating enzyme E2 N like (gene/pseudogene)(UBE2NL) | Homo sapiens |
| 63893 | UBE2O | ubiquitin conjugating enzyme E2 O(UBE2O) | Homo sapiens |
| 55585 | UBE2Q1 | ubiquitin conjugating enzyme E2 Q1(UBE2Q1) | Homo sapiens |
| 92912 | UBE2Q2 | ubiquitin conjugating enzyme E2 Q2(UBE2Q2) | Homo sapiens |
| 134111 | UBE2QL1 | ubiquitin conjugating enzyme E2 Q family like 1(UBE2QL1) | Homo sapiens |
| 54926 | UBE2R2 | ubiquitin conjugating enzyme E2 R2(UBE2R2) | Homo sapiens |
| 27338 | UBE2S | ubiquitin conjugating enzyme E2 S(UBE2S) | Homo sapiens |
| 29089 | UBE2T | ubiquitin conjugating enzyme E2 T(UBE2T) | Homo sapiens |
| 148581 | UBE2U | ubiquitin conjugating enzyme E2 U (putative)(UBE2U) | Homo sapiens |
| 7335 | UBE2V1 | ubiquitin conjugating enzyme E2 V1(UBE2V1) | Homo sapiens |
| 7336 | UBE2V2 | ubiquitin conjugating enzyme E2 V2(UBE2V2) | Homo sapiens |
| 55284 | UBE2W | ubiquitin conjugating enzyme E2 W (putative)(UBE2W) | Homo sapiens |
| 65264 | UBE2Z | ubiquitin conjugating enzyme E2 Z(UBE2Z) | Homo sapiens |
| 83734 | ATG10 | autophagy related 10(ATG10) | Homo sapiens |
| 64422 | ATG3 | autophagy related 3(ATG3) | Homo sapiens |
| 57448 | BIRC6 | baculoviral IAP repeat containing 6(BIRC6) | Homo sapiens |
| 997 | CDC34 | cell division cycle 34(CDC34) | Homo sapiens |
| E3s |  |  |  |
| 80325 | ABTB1 | ankyrin repeat and BTB domain containing 1(ABTB1) | Homo sapiens |
| 25841 | ABTB2 | ankyrin repeat and BTB domain containing 2(ABTB2) | Homo sapiens |
| 267 | AMFR | autocrine motility factor receptor(AMFR) | Homo sapiens |
| 51529 | ANAPC11 | anaphase promoting complex subunit 11(ANAPC11) | Homo sapiens |
| 51479 | ANKFY1 | ankyrin repeat and FYVE domain containing 1(ANKFY1) | Homo sapiens |
| 54467 | ANKIB1 | ankyrin repeat and IBR domain containing 1(ANKIB1) | Homo sapiens |
| 93550 | ZFAND4 (ANUBL1) | zinc finger AN1-type containing 4 (ZFAND4) | Homo sapiens |
| 25820 | ARIH1 | ariadne RBR E3 ubiquitin protein ligase 1(ARIH1) | Homo sapiens |
| 10425 | ARIH2 | ariadne RBR E3 ubiquitin protein ligase 2(ARIH2) | Homo sapiens |
| 79798 | ARMC5 | armadillo repeat containing 5(ARMC5) | Homo sapiens |
| 136371 | ASB10 | ankyrin repeat and SOCS box containing 10(ASB10) | Homo sapiens |
| 140456 | ASB11 | ankyrin repeat and SOCS box containing 11(ASB11) | Homo sapiens |
| 79754 | ASB13 | ankyrin repeat and SOCS box containing 13(ASB13) | Homo sapiens |
| 142686 | ASB14 | ankyrin repeat and SOCS box containing 14(ASB14) | Homo sapiens |
| 142685 | ASB15 | ankyrin repeat and SOCS box containing 15(ASB15) | Homo sapiens |
| 92591 | ASB16 | ankyrin repeat and SOCS box containing 16(ASB16) | Homo sapiens |
| 127247 | ASB17 | ankyrin repeat and SOCS box containing 17(ASB17) | Homo sapiens |
| 401036 | ASB18 | ankyrin repeat and SOCS box containing 18(ASB18) | Homo sapiens |
| 51676 | ASB2 | ankyrin repeat and SOCS box containing 2(ASB2) | Homo sapiens |
| 51130 | ASB3 | ankyrin repeat and SOCS box containing 3(ASB3) | Homo sapiens |
| 51666 | ASB4 | ankyrin repeat and SOCS box containing 4(ASB4) | Homo sapiens |
| 140458 | ASB5 | ankyrin repeat and SOCS box containing 5(ASB5) | Homo sapiens |
| 140459 | ASB6 | ankyrin repeat and SOCS box containing 6(ASB6) | Homo sapiens |
| 140460 | ASB7 | ankyrin repeat and SOCS box containing 7(ASB7) | Homo sapiens |
| 140461 | ASB8 | ankyrin repeat and SOCS box containing 8(ASB8) | Homo sapiens |
| 140462 | ASB9 | ankyrin repeat and SOCS box containing 9(ASB9) | Homo sapiens |
| 546 | ATRX | ATRX, chromatin remodeler(ATRX) | Homo sapiens |
| 571 | BACH1 | BTB domain and CNC homolog 1(BACH1) | Homo sapiens |
| 60468 | BACH2 | BTB domain and CNC homolog 2(BACH2) | Homo sapiens |
| 580 | BARD1 | BRCA1 associated RING domain 1(BARD1) | Homo sapiens |
| 604 | BCL6 | B-cell CLL/lymphoma 6(BCL6) | Homo sapiens |
| 255877 | BCL6B | B-cell CLL/lymphoma 6B(BCL6B) | Homo sapiens |
| 51283 | BFAR | bifunctional apoptosis regulator(BFAR) | Homo sapiens |
| 329 | BIRC2 | baculoviral IAP repeat containing 2(BIRC2) | Homo sapiens |
| 330 | BIRC3 | baculoviral IAP repeat containing 3(BIRC3) | Homo sapiens |
| 79444 | BIRC7 | baculoviral IAP repeat containing 7(BIRC7) | Homo sapiens |
| 112401 | BIRC8 | baculoviral IAP repeat containing 8(BIRC8) | Homo sapiens |
| 648 | BMI1 | BMI1 proto-oncogene, polycomb ring finger(BMI1) | Homo sapiens |
| 8315 | BRAP | BRCA1 associated protein(BRAP) | Homo sapiens |
| 672 | BRCA1 | BRCA1, DNA repair associated(BRCA1) | Homo sapiens |
| 53339 | BTBD1 | BTB domain containing 1(BTBD1) | Homo sapiens |
| 84280 | BTBD10 | BTB domain containing 10(BTBD10) | Homo sapiens |
| 121551 | BTBD11 | BTB domain containing 11(BTBD11) | Homo sapiens |
| 84464 | SLX4 (BTBD12) | SLX4 structure-specific endonuclease subunit (SLX4) | Homo sapiens |
| 388419 | BTBD17 | BTB domain containing 17(BTBD17) | Homo sapiens |
| 55643 | BTBD2 | BTB domain containing 2(BTBD2) | Homo sapiens |
| 22903 | BTBD3 | BTB domain containing 3(BTBD3) | Homo sapiens |
| 90135 | BTBD6 | BTB domain containing 6(BTBD6) | Homo sapiens |
| 55727 | BTBD7 | BTB domain containing 7(BTBD7) | Homo sapiens |
| 284697 | BTBD8 | BTB domain containing 8(BTBD8) | Homo sapiens |
| 114781 | BTBD9 | BTB domain containing 9(BTBD9) | Homo sapiens |
| 8945 | BTRC | beta-transducin repeat containing E3 ubiquitin protein ligase(BTRC) | Homo sapiens |
| 283450 | HECTD4 (C12orf51) | HECT domain E3 ubiquitin protein ligase 4(HECTD4) | Homo sapiens |
| 64207 | IRF2BPL (C14orf4) | interferon regulatory factor 2 binding protein like (IRF2BPL) | Homo sapiens |
| 867 | CBL | Cbl proto-oncogene(CBL) | Homo sapiens |
| 868 | CBLB | Cbl proto-oncogene B(CBLB) | Homo sapiens |
| 23624 | CBLC | Cbl proto-oncogene C(CBLC) | Homo sapiens |
| 79872 | CBLL1 | Cbl proto-oncogene like 1(CBLL1) | Homo sapiens |
| 881 | CCIN | calicin(CCIN) | Homo sapiens |
| 57820 | CCNB1IP1 | cyclin B1 interacting protein 1(CCNB1IP1) | Homo sapiens |
| 899 | CCNF | cyclin F(CCNF) | Homo sapiens |
| 10668 | CGRRF1 | cell growth regulator with ring finger domain 1(CGRRF1) | Homo sapiens |
| 55743 | CHFR | checkpoint with forkhead and ring finger domains(CHFR) | Homo sapiens |
| 1154 | CISH | cytokine inducible SH2 containing protein(CISH) | Homo sapiens |
| 4850 | CNOT4 | CCR4-NOT transcription complex subunit 4(CNOT4) | Homo sapiens |
| 29894 | CPSF1 | cleavage and polyadenylation specific factor 1(CPSF1) | Homo sapiens |
| 23113 | CUL9 | cullin 9(CUL9) | Homo sapiens |
| 149095 | DCST1 | DC-STAMP domain containing 1(DCST1) | Homo sapiens |
| 1642 | DDB1 | damage specific DNA binding protein 1(DDB1) | Homo sapiens |
| 8193 | DPF1 | double PHD fingers 1(DPF1) | Homo sapiens |
| 1840 | DTX1 | deltex E3 ubiquitin ligase 1(DTX1) | Homo sapiens |
| 113878 | DTX2 | deltex E3 ubiquitin ligase 2(DTX2) | Homo sapiens |
| 196403 | DTX3 | deltex E3 ubiquitin ligase 3(DTX3) | Homo sapiens |
| 151636 | DTX3L | deltex E3 ubiquitin ligase 3L(DTX3L) | Homo sapiens |
| 23220 | DTX4 | deltex E3 ubiquitin ligase 4(DTX4) | Homo sapiens |
| 9666 | DZIP3 | DAZ interacting zinc finger protein 3(DZIP3) | Homo sapiens |
| 345930 | ECT2L | epithelial cell transforming 2 like(ECT2L) | Homo sapiens |
| 8507 | ENC1 | ectodermal-neural cortex 1(ENC1) | Homo sapiens |
| 55120 | FANCL | Fanconi anemia complementation group L(FANCL) | Homo sapiens |
| 54850 | FBXL12 | F-box and leucine rich repeat protein 12(FBXL12) | Homo sapiens |
| 222235 | FBXL13 | F-box and leucine rich repeat protein 13(FBXL13) | Homo sapiens |
| 144699 | FBXL14 | F-box and leucine rich repeat protein 14(FBXL14) | Homo sapiens |
| 79176 | FBXL15 | F-box and leucine rich repeat protein 15(FBXL15) | Homo sapiens |
| 146330 | FBXL16 | F-box and leucine rich repeat protein 16(FBXL16) | Homo sapiens |
| 80028 | FBXL18 | F-box and leucine rich repeat protein 18(FBXL18) | Homo sapiens |
| 25827 | FBXL2 | F-box and leucine rich repeat protein 2(FBXL2) | Homo sapiens |
| 84961 | FBXL20 | F-box and leucine rich repeat protein 20(FBXL20) | Homo sapiens |
| 26224 | FBXL3 | F-box and leucine rich repeat protein 3(FBXL3) | Homo sapiens |
| 26235 | FBXL4 | F-box and leucine rich repeat protein 4(FBXL4) | Homo sapiens |
| 26234 | FBXL5 | F-box and leucine rich repeat protein 5(FBXL5) | Homo sapiens |
| 26233 | FBXL6 | F-box and leucine rich repeat protein 6(FBXL6) | Homo sapiens |
| 23194 | FBXL7 | F-box and leucine rich repeat protein 7(FBXL7) | Homo sapiens |
| 55336 | FBXL8 | F-box and leucine rich repeat protein 8(FBXL8) | Homo sapiens |
| 26267 | FBXO10 | F-box protein 10(FBXO10) | Homo sapiens |
| 80204 | FBXO11 | F-box protein 11(FBXO11) | Homo sapiens |
| 201456 | FBXO15 | F-box protein 15(FBXO15) | Homo sapiens |
| 157574 | FBXO16 | F-box protein 16(FBXO16) | Homo sapiens |
| 115290 | FBXO17 | F-box protein 17(FBXO17) | Homo sapiens |
| 84893 | FBXO18 | F-box protein, helicase, 18(FBXO18) | Homo sapiens |
| 26232 | FBXO2 | F-box protein 2(FBXO2) | Homo sapiens |
| 23014 | FBXO21 | F-box protein 21(FBXO21) | Homo sapiens |
| 26263 | FBXO22 | F-box protein 22(FBXO22) | Homo sapiens |
| 26261 | FBXO24 | F-box protein 24(FBXO24) | Homo sapiens |
| 26260 | FBXO25 | F-box protein 25(FBXO25) | Homo sapiens |
| 126433 | FBXO27 | F-box protein 27(FBXO27) | Homo sapiens |
| 23219 | FBXO28 | F-box protein 28(FBXO28) | Homo sapiens |
| 26273 | FBXO3 | F-box protein 3(FBXO3) | Homo sapiens |
| 84085 | FBXO30 | F-box protein 30(FBXO30) | Homo sapiens |
| 79791 | FBXO31 | F-box protein 31(FBXO31) | Homo sapiens |
| 114907 | FBXO32 | F-box protein 32(FBXO32) | Homo sapiens |
| 254170 | FBXO33 | F-box protein 33(FBXO33) | Homo sapiens |
| 55030 | FBXO34 | F-box protein 34(FBXO34) | Homo sapiens |
| 130888 | FBXO36 | F-box protein 36(FBXO36) | Homo sapiens |
| 81545 | FBXO38 | F-box protein 38(FBXO38) | Homo sapiens |
| 26272 | FBXO4 | F-box protein 4(FBXO4) | Homo sapiens |
| 51725 | FBXO40 | F-box protein 40(FBXO40) | Homo sapiens |
| 150726 | FBXO41 | F-box protein 41(FBXO41) | Homo sapiens |
| 54455 | FBXO42 | F-box protein 42(FBXO42) | Homo sapiens |
| 93611 | FBXO44 | F-box protein 44(FBXO44) | Homo sapiens |
| 200933 | FBXO45 | F-box protein 45(FBXO45) | Homo sapiens |
| 23403 | FBXO46 | F-box protein 46(FBXO46) | Homo sapiens |
| 26271 | FBXO5 | F-box protein 5(FBXO5) | Homo sapiens |
| 26270 | FBXO6 | F-box protein 6(FBXO6) | Homo sapiens |
| 25793 | FBXO7 | F-box protein 7(FBXO7) | Homo sapiens |
| 26269 | FBXO8 | F-box protein 8(FBXO8) | Homo sapiens |
| 26268 | FBXO9 | F-box protein 9(FBXO9) | Homo sapiens |
| 23291 | FBXW11 | F-box and WD repeat domain containing 11(FBXW11) | Homo sapiens |
| 26190 | FBXW2 | F-box and WD repeat domain containing 2(FBXW2) | Homo sapiens |
| 6468 | FBXW4 | F-box and WD repeat domain containing 4(FBXW4) | Homo sapiens |
| 54461 | FBXW5 | F-box and WD repeat domain containing 5(FBXW5) | Homo sapiens |
| 55294 | FBXW7 | F-box and WD repeat domain containing 7(FBXW7) | Homo sapiens |
| 26259 | FBXW8 | F-box and WD repeat domain containing 8(FBXW8) | Homo sapiens |
| 55632 | G2E3 | G2/M-phase specific E3 ubiquitin protein ligase(G2E3) | Homo sapiens |
| 8139 | GAN | gigaxonin(GAN) | Homo sapiens |
| 64395 | GMCL1 | germ cell-less, spermatogenesis associated 1(GMCL1) | Homo sapiens |
| 2966 | GTF2H2 | general transcription factor IIH subunit 2(GTF2H2) | Homo sapiens |
| 64412 | GZF1 | GDNF inducible zinc finger protein 1(GZF1) | Homo sapiens |
| 57531 | HACE1 | HECT domain and ankyrin repeat containing E3 ubiquitin protein ligase 1(HACE1) | Homo sapiens |
| 25831 | HECTD1 | HECT domain E3 ubiquitin protein ligase 1(HECTD1) | Homo sapiens |
| 143279 | HECTD2 | HECT domain E3 ubiquitin protein ligase 2(HECTD2) | Homo sapiens |
| 79654 | HECTD3 | HECT domain E3 ubiquitin protein ligase 3(HECTD3) | Homo sapiens |
| 23072 | HECW1 | HECT, C2 and WW domain containing E3 ubiquitin protein ligase 1(HECW1) | Homo sapiens |
| 57520 | HECW2 | HECT, C2 and WW domain containing E3 ubiquitin protein ligase 2(HECW2) | Homo sapiens |
| 8925 | HERC1 | HECT and RLD domain containing E3 ubiquitin protein ligase family member 1(HERC1) | Homo sapiens |
| 8924 | HERC2 | HECT and RLD domain containing E3 ubiquitin protein ligase 2(HERC2) | Homo sapiens |
| 8916 | HERC3 | HECT and RLD domain containing E3 ubiquitin protein ligase 3(HERC3) | Homo sapiens |
| 26091 | HERC4 | HECT and RLD domain containing E3 ubiquitin protein ligase 4(HERC4) | Homo sapiens |
| 51191 | HERC5 | HECT and RLD domain containing E3 ubiquitin protein ligase 5(HERC5) | Homo sapiens |
| 55008 | HERC6 | HECT and RLD domain containing E3 ubiquitin protein ligase family member 6(HERC6) | Homo sapiens |
| 3090 | HIC1 | HIC ZBTB transcriptional repressor 1(HIC1) | Homo sapiens |
| 23119 | HIC2 | HIC ZBTB transcriptional repressor 2(HIC2) | Homo sapiens |
| 6596 | HLTF | helicase like transcription factor(HLTF) | Homo sapiens |
| 10075 | HUWE1 | HECT, UBA and WWE domain containing 1, E3 ubiquitin protein ligase(HUWE1) | Homo sapiens |
| 25998 | IBTK | inhibitor of Bruton tyrosine kinase(IBTK) | Homo sapiens |
| 3652 | IPP | intracisternal A particle-promoted polypeptide(IPP) | Homo sapiens |
| 83737 | ITCH | itchy E3 ubiquitin protein ligase(ITCH) | Homo sapiens |
| 10625 | IVNS1ABP | influenza virus NS1A binding protein(IVNS1ABP) | Homo sapiens |
| 10324 | KLHL41 (KBTBD10) | kelch like family member 41(KLHL41) | Homo sapiens |
| 9920 | KBTBD11 | kelch repeat and BTB domain containing 11(KBTBD11) | Homo sapiens |
| 166348 | KBTBD12 | kelch repeat and BTB domain containing 12(KBTBD12) | Homo sapiens |
| 25948 | KBTBD2 | kelch repeat and BTB domain containing 2(KBTBD2) | Homo sapiens |
| 143879 | KBTBD3 | kelch repeat and BTB domain containing 3(KBTBD3) | Homo sapiens |
| 55709 | KBTBD4 | kelch repeat and BTB domain containing 4(KBTBD4) | Homo sapiens |
| 131377 | KLHL40 (KBTBD5) | kelch like family member 40(KLHL40) | Homo sapiens |
| 89890 | KBTBD6 | kelch repeat and BTB domain containing 6(KBTBD6) | Homo sapiens |
| 84078 | KBTBD7 | kelch repeat and BTB domain containing 7(KBTBD7) | Homo sapiens |
| 84541 | KBTBD8 | kelch repeat and BTB domain containing 8(KBTBD8) | Homo sapiens |
| 3736 | KCNA1 | potassium voltage-gated channel subfamily A member 1(KCNA1) | Homo sapiens |
| 3744 | KCNA10 | potassium voltage-gated channel subfamily A member 10(KCNA10) | Homo sapiens |
| 3737 | KCNA2 | potassium voltage-gated channel subfamily A member 2(KCNA2) | Homo sapiens |
| 3738 | KCNA3 | potassium voltage-gated channel subfamily A member 3(KCNA3) | Homo sapiens |
| 3739 | KCNA4 | potassium voltage-gated channel subfamily A member 4(KCNA4) | Homo sapiens |
| 3741 | KCNA5 | potassium voltage-gated channel subfamily A member 5(KCNA5) | Homo sapiens |
| 3742 | KCNA6 | potassium voltage-gated channel subfamily A member 6(KCNA6) | Homo sapiens |
| 3743 | KCNA7 | potassium voltage-gated channel subfamily A member 7(KCNA7) | Homo sapiens |
| 3745 | KCNB1 | potassium voltage-gated channel subfamily B member 1(KCNB1) | Homo sapiens |
| 9312 | KCNB2 | potassium voltage-gated channel subfamily B member 2(KCNB2) | Homo sapiens |
| 3746 | KCNC1 | potassium voltage-gated channel subfamily C member 1(KCNC1) | Homo sapiens |
| 3747 | KCNC2 | potassium voltage-gated channel subfamily C member 2(KCNC2) | Homo sapiens |
| 3748 | KCNC3 | potassium voltage-gated channel subfamily C member 3(KCNC3) | Homo sapiens |
| 3749 | KCNC4 | potassium voltage-gated channel subfamily C member 4(KCNC4) | Homo sapiens |
| 3750 | KCND1 | potassium voltage-gated channel subfamily D member 1(KCND1) | Homo sapiens |
| 3751 | KCND2 | potassium voltage-gated channel subfamily D member 2(KCND2) | Homo sapiens |
| 3752 | KCND3 | potassium voltage-gated channel subfamily D member 3(KCND3) | Homo sapiens |
| 3755 | KCNG1 | potassium voltage-gated channel modifier subfamily G member 1(KCNG1) | Homo sapiens |
| 170850 | KCNG3 | potassium voltage-gated channel modifier subfamily G member 3(KCNG3) | Homo sapiens |
| 283518 | KCNRG | potassium channel regulator(KCNRG) | Homo sapiens |
| 3787 | KCNS1 | potassium voltage-gated channel modifier subfamily S member 1(KCNS1) | Homo sapiens |
| 3788 | KCNS2 | potassium voltage-gated channel modifier subfamily S member 2(KCNS2) | Homo sapiens |
| 3790 | KCNS3 | potassium voltage-gated channel modifier subfamily S member 3(KCNS3) | Homo sapiens |
| 27012 | KCNV1 | potassium voltage-gated channel modifier subfamily V member 1(KCNV1) | Homo sapiens |
| 284252 | KCTD1 | potassium channel tetramerization domain containing 1(KCTD1) | Homo sapiens |
| 83892 | KCTD10 | potassium channel tetramerization domain containing 10(KCTD10) | Homo sapiens |
| 115207 | KCTD12 | potassium channel tetramerization domain containing 12(KCTD12) | Homo sapiens |
| 253980 | KCTD13 | potassium channel tetramerization domain containing 13(KCTD13) | Homo sapiens |
| 65987 | KCTD14 | potassium channel tetramerization domain containing 14(KCTD14) | Homo sapiens |
| 79047 | KCTD15 | potassium channel tetramerization domain containing 15(KCTD15) | Homo sapiens |
| 57528 | KCTD16 | potassium channel tetramerization domain containing 16(KCTD16) | Homo sapiens |
| 79734 | KCTD17 | potassium channel tetramerization domain containing 17(KCTD17) | Homo sapiens |
| 23510 | KCTD2 | potassium channel tetramerization domain containing 2(KCTD2) | Homo sapiens |
| 222658 | KCTD20 | potassium channel tetramerization domain containing 20(KCTD20) | Homo sapiens |
| 283219 | KCTD21 | potassium channel tetramerization domain containing 21(KCTD21) | Homo sapiens |
| 51133 | KCTD3 | potassium channel tetramerization domain containing 3(KCTD3) | Homo sapiens |
| 386618 | KCTD4 | potassium channel tetramerization domain containing 4(KCTD4) | Homo sapiens |
| 54442 | KCTD5 | potassium channel tetramerization domain containing 5(KCTD5) | Homo sapiens |
| 200845 | KCTD6 | potassium channel tetramerization domain containing 6(KCTD6) | Homo sapiens |
| 154881 | KCTD7 | potassium channel tetramerization domain containing 7(KCTD7) | Homo sapiens |
| 154881 | KCTD7 | potassium channel tetramerization domain containing 7(KCTD7) | Homo sapiens |
| 386617 | KCTD8 | potassium channel tetramerization domain containing 8(KCTD8) | Homo sapiens |
| 54793 | KCTD9 | potassium channel tetramerization domain containing 9(KCTD9) | Homo sapiens |
| 22992 | KDM2A | lysine demethylase 2A(KDM2A) | Homo sapiens |
| 84678 | KDM2B | lysine demethylase 2B(KDM2B) | Homo sapiens |
| 9817 | KEAP1 | kelch like ECH associated protein 1(KEAP1) | Homo sapiens |
| 9870 | AREL1 (KIAA0317) | apoptosis resistant E3 ubiquitin protein ligase 1(AREL1) | Homo sapiens |
| 57542 | KLHL42 (KLHDC5) | kelch like family member 42(KLHL42) | Homo sapiens |
| 57626 | KLHL1 | kelch like family member 1(KLHL1) | Homo sapiens |
| 317719 | KLHL10 | kelch like family member 10(KLHL10) | Homo sapiens |
| 55175 | KLHL11 | kelch like family member 11(KLHL11) | Homo sapiens |
| 59349 | KLHL12 | kelch like family member 12(KLHL12) | Homo sapiens |
| 90293 | KLHL13 | kelch like family member 13(KLHL13) | Homo sapiens |
| 57565 | KLHL14 | kelch like family member 14(KLHL14) | Homo sapiens |
| 80311 | KLHL15 | kelch like family member 15(KLHL15) | Homo sapiens |
| 339451 | KLHL17 | kelch like family member 17(KLHL17) | Homo sapiens |
| 23276 | KLHL18 | kelch like family member 18(KLHL18) | Homo sapiens |
| 27252 | KLHL20 | kelch like family member 20(KLHL20) | Homo sapiens |
| 9903 | KLHL21 | kelch like family member 21(KLHL21) | Homo sapiens |
| 84861 | KLHL22 | kelch like family member 22(KLHL22) | Homo sapiens |
| 151230 | KLHL23 | kelch like family member 23(KLHL23) | Homo sapiens |
| 54800 | KLHL24 | kelch like family member 24(KLHL24) | Homo sapiens |
| 64410 | KLHL25 | kelch like family member 25(KLHL25) | Homo sapiens |
| 55295 | KLHL26 | kelch like family member 26(KLHL26) | Homo sapiens |
| 54813 | KLHL28 | kelch like family member 28(KLHL28) | Homo sapiens |
| 114818 | KLHL29 | kelch like family member 29(KLHL29) | Homo sapiens |
| 26249 | KLHL3 | kelch like family member 3(KLHL3) | Homo sapiens |
| 377007 | KLHL30 | kelch like family member 30(KLHL30) | Homo sapiens |
| 401265 | KLHL31 | kelch like family member 31(KLHL31) | Homo sapiens |
| 114792 | KLHL32 | kelch like family member 32(KLHL32) | Homo sapiens |
| 123103 | KLHL33 | kelch like family member 33(KLHL33) | Homo sapiens |
| 257240 | KLHL34 | kelch like family member 34(KLHL34) | Homo sapiens |
| 79786 | KLHL36 | kelch like family member 36(KLHL36) | Homo sapiens |
| 340359 | KLHL38 | kelch like family member 38(KLHL38) | Homo sapiens |
| 56062 | KLHL4 | kelch like family member 4(KLHL4) | Homo sapiens |
| 51088 | KLHL5 | kelch like family member 5(KLHL5) | Homo sapiens |
| 89857 | KLHL6 | kelch like family member 6(KLHL6) | Homo sapiens |
| 55975 | KLHL7 | kelch like family member 7(KLHL7) | Homo sapiens |
| 57563 | KLHL8 | kelch like family member 8(KLHL8) | Homo sapiens |
| 55958 | KLHL9 | kelch like family member 9(KLHL9) | Homo sapiens |
| 3856 | KRT8 | keratin 8(KRT8) | Homo sapiens |
| 3959 | LGALS3BP | galectin 3 binding protein(LGALS3BP) | Homo sapiens |
| 84708 | LNX1 | ligand of numb-protein X 1(LNX1) | Homo sapiens |
| 222484 | LNX2 | ligand of numb-protein X 2(LNX2) | Homo sapiens |
| 91694 | LONRF1 | LON peptidase N-terminal domain and ring finger 1(LONRF1) | Homo sapiens |
| 164832 | LONRF2 | LON peptidase N-terminal domain and ring finger 2(LONRF2) | Homo sapiens |
| 79836 | LONRF3 | LON peptidase N-terminal domain and ring finger 3(LONRF3) | Homo sapiens |
| 26231 | LRRC29 | leucine rich repeat containing 29(LRRC29) | Homo sapiens |
| 90678 | LRSAM1 | leucine rich repeat and sterile alpha motif containing 1(LRSAM1) | Homo sapiens |
| 8216 | LZTR1 | leucine zipper like transcription regulator 1(LZTR1) | Homo sapiens |
| 4214 | MAP3K1 | mitogen-activated protein kinase kinase kinase 1(MAP3K1) | Homo sapiens |
| 55016 | MARCH1 | membrane associated ring-CH-type finger 1(MARCH1) | Homo sapiens |
| 162333 | MARCH10 | membrane associated ring-CH-type finger 10(MARCH10) | Homo sapiens |
| 441061 | MARCH11 | membrane associated ring-CH-type finger 11(MARCH11) | Homo sapiens |
| 51257 | MARCH2 | membrane associated ring-CH-type finger 2(MARCH2) | Homo sapiens |
| 115123 | MARCH3 | membrane associated ring-CH-type finger 3(MARCH3) | Homo sapiens |
| 57574 | MARCH4 | membrane associated ring-CH-type finger 4(MARCH4) | Homo sapiens |
| 54708 | MARCH5 | membrane associated ring-CH-type finger 5(MARCH5) | Homo sapiens |
| 10299 | MARCH6 | membrane associated ring-CH-type finger 6(MARCH6) | Homo sapiens |
| 64844 | MARCH7 | membrane associated ring-CH-type finger 7(MARCH7) | Homo sapiens |
| 220972 | MARCH8 | membrane associated ring-CH-type finger 8(MARCH8) | Homo sapiens |
| 92979 | MARCH9 | membrane associated ring-CH-type finger 9(MARCH9) | Homo sapiens |
| 4193 | MDM2 | MDM2 proto-oncogene(MDM2) | Homo sapiens |
| 4194 | MDM4 | MDM4, p53 regulator(MDM4) | Homo sapiens |
| 92312 | MEX3A | mex-3 RNA binding family member A(MEX3A) | Homo sapiens |
| 84206 | MEX3B | mex-3 RNA binding family member B(MEX3B) | Homo sapiens |
| 51320 | MEX3C | mex-3 RNA binding family member C(MEX3C) | Homo sapiens |
| 399664 | MEX3D | mex-3 RNA binding family member D(MEX3D) | Homo sapiens |
| 196872 | LINC00638 (MGC23270) | long intergenic non-protein coding RNA 638 | Homo sapiens |
| 23295 | MGRN1 | mahogunin ring finger 1(MGRN1) | Homo sapiens |
| 57534 | MIB1 | mindbomb E3 ubiquitin protein ligase 1(MIB1) | Homo sapiens |
| 142678 | MIB2 | mindbomb E3 ubiquitin protein ligase 2(MIB2) | Homo sapiens |
| 4281 | MID1 | midline 1(MID1) | Homo sapiens |
| 11043 | MID2 | midline 2(MID2) | Homo sapiens |
| 23608 | MKRN1 | makorin ring finger protein 1(MKRN1) | Homo sapiens |
| 23609 | MKRN2 | makorin ring finger protein 2(MKRN2) | Homo sapiens |
| 7681 | MKRN3 | makorin ring finger protein 3(MKRN3) | Homo sapiens |
| 4331 | MNAT1 | MNAT1, CDK activating kinase assembly factor(MNAT1) | Homo sapiens |
| 55167 | MSL2 | male-specific lethal 2 homolog (Drosophila)(MSL2) | Homo sapiens |
| 79594 | MUL1 | mitochondrial E3 ubiquitin protein ligase 1(MUL1) | Homo sapiens |
| 23077 | MYCBP2 | MYC binding protein 2, E3 ubiquitin protein ligase(MYCBP2) | Homo sapiens |
| 29116 | MYLIP | myosin regulatory light chain interacting protein(MYLIP) | Homo sapiens |
| 55892 | MYNN | myoneurin(MYNN) | Homo sapiens |
| 112939 | NACC1 | nucleus accumbens associated 1(NACC1) | Homo sapiens |
| 138151 | NACC2 | NACC family member 2(NACC2) | Homo sapiens |
| 4734 | NEDD4 | neural precursor cell expressed, developmentally down-regulated 4, E3 ubiquitin protein ligase(NEDD4) | Homo sapiens |
| 23327 | NEDD4L | neural precursor cell expressed, developmentally down-regulated 4-like, E3 ubiquitin protein ligase(NEDD4L) | Homo sapiens |
| 9148 | NEURL1(NEURL) | neuralized E3 ubiquitin protein ligase 1(NEURL1) | Homo sapiens |
| 140825 | NEURL2 | neuralized E3 ubiquitin protein ligase 2(NEURL2) | Homo sapiens |
| 93082 | NEURL3 | neuralized E3 ubiquitin protein ligase 3(NEURL3) | Homo sapiens |
| 4799 | NFX1 | nuclear transcription factor, X-box binding 1(NFX1) | Homo sapiens |
| 152518 | NFXL1 | nuclear transcription factor, X-box binding like 1(NFXL1) | Homo sapiens |
| 378884 | NHLRC1 | NHL repeat containing E3 ubiquitin protein ligase 1(NHLRC1) | Homo sapiens |
| 51070 | NOSIP | nitric oxide synthase interacting protein(NOSIP) | Homo sapiens |
| 197370 | NSMCE1 | NSE1 homolog, SMC5-SMC6 complex component(NSMCE1) | Homo sapiens |
| 28962 | OSTM1 | osteopetrosis associated transmembrane protein 1(OSTM1) | Homo sapiens |
| 161725 | OTUD7A | OTU deubiquitinase 7A(OTUD7A) | Homo sapiens |
| 56957 | OTUD7B | OTU deubiquitinase 7B(OTUD7B) | Homo sapiens |
| 5071 | PARK2 | parkin RBR E3 ubiquitin protein ligase(PARK2) | Homo sapiens |
| 23598 | PATZ1 | POZ/BTB and AT hook containing zinc finger 1(PATZ1) | Homo sapiens |
| 84759 | PCGF1 | polycomb group ring finger 1(PCGF1) | Homo sapiens |
| 7703 | PCGF2 | polycomb group ring finger 2(PCGF2) | Homo sapiens |
| 10336 | PCGF3 | polycomb group ring finger 3(PCGF3) | Homo sapiens |
| 84333 | PCGF5 | polycomb group ring finger 5(PCGF5) | Homo sapiens |
| 84108 | PCGF6 | polycomb group ring finger 6(PCGF6) | Homo sapiens |
| 23024 | PDZRN3 | PDZ domain containing ring finger 3(PDZRN3) | Homo sapiens |
| 29951 | PDZRN4 | PDZ domain containing ring finger 4(PDZRN4) | Homo sapiens |
| 5192 | PEX10 | peroxisomal biogenesis factor 10(PEX10) | Homo sapiens |
| 5193 | PEX12 | peroxisomal biogenesis factor 12(PEX12) | Homo sapiens |
| 5828 | PEX2 | peroxisomal biogenesis factor 2(PEX2) | Homo sapiens |
| 51533 | PHF7 | PHD finger protein 7(PHF7) | Homo sapiens |
| 57661 | PHRF1 | PHD and ring finger domains 1(PHRF1) | Homo sapiens |
| 64219 | PJA1 | praja ring finger ubiquitin ligase 1(PJA1) | Homo sapiens |
| 9867 | PJA2 | praja ring finger ubiquitin ligase 2(PJA2) | Homo sapiens |
| 5371 | PML | promyelocytic leukemia(PML) | Homo sapiens |
| 23759 | PPIL2 | peptidylprolyl isomerase like 2(PPIL2) | Homo sapiens |
| 27339 | PRPF19 | pre-mRNA processing factor 19(PRPF19) | Homo sapiens |
| 142684 | RAB40A | RAB40A, member RAS oncogene family(RAB40A) | Homo sapiens |
| 282808 | RAB40AL | RAB40A, member RAS oncogene family-like(RAB40AL) | Homo sapiens |
| 10966 | RAB40B | RAB40B, member RAS oncogene family(RAB40B) | Homo sapiens |
| 57799 | RAB40C | RAB40C, member RAS oncogene family(RAB40C) | Homo sapiens |
| 27342 | RABGEF1 | RAB guanine nucleotide exchange factor 1(RABGEF1) | Homo sapiens |
| 56852 | RAD18 | RAD18, E3 ubiquitin protein ligase(RAD18) | Homo sapiens |
| 5896 | RAG1 | recombination activating 1(RAG1) | Homo sapiens |
| 5913 | RAPSN | receptor associated protein of the synapse(RAPSN) | Homo sapiens |
| 5930 | RBBP6 | RB binding protein 6, ubiquitin ligase(RBBP6) | Homo sapiens |
| 10616 | RBCK1 | RANBP2-type and C3HC4-type zinc finger containing 1(RBCK1) | Homo sapiens |
| 9978 | RBX1 | ring-box 1(RBX1) | Homo sapiens |
| 149041 | RC3H1 | ring finger and CCCH-type domains 1(RC3H1) | Homo sapiens |
| 54542 | RC3H2 | ring finger and CCCH-type domains 2(RC3H2) | Homo sapiens |
| 55213 | RCBTB1 | RCC1 and BTB domain containing protein 1(RCBTB1) | Homo sapiens |
| 1102 | RCBTB2 | RCC1 and BTB domain containing protein 2(RCBTB2) | Homo sapiens |
| 25898 | RCHY1 | ring finger and CHY zinc finger domain containing 1(RCHY1) | Homo sapiens |
| 117584 | RFFL | ring finger and FYVE like domain containing E3 ubiquitin protein ligase(RFFL) | Homo sapiens |
| 5988 | RFPL1 | ret finger protein like 1(RFPL1) | Homo sapiens |
| 10738 | RFPL3 | ret finger protein like 3(RFPL3) | Homo sapiens |
| 342931 | RFPL4A | ret finger protein like 4A(RFPL4A) | Homo sapiens |
| 442247 | RFPL4B | ret finger protein like 4B(RFPL4B) | Homo sapiens |
| 64326 | RFWD2 | ring finger and WD repeat domain 2(RFWD2) | Homo sapiens |
| 55159 | RFWD3 | ring finger and WD repeat domain 3(RFWD3) | Homo sapiens |
| 9886 | RHOBTB1 | Rho related BTB domain containing 1(RHOBTB1) | Homo sapiens |
| 23221 | RHOBTB2 | Rho related BTB domain containing 2(RHOBTB2) | Homo sapiens |
| 22836 | RHOBTB3 | Rho related BTB domain containing 3(RHOBTB3) | Homo sapiens |
| 6015 | RING1 | ring finger protein 1(RING1) | Homo sapiens |
| 51132 | RLIM | ring finger protein, LIM domain interacting(RLIM) | Homo sapiens |
| 9921 | RNF10 | ring finger protein 10(RNF10) | Homo sapiens |
| 7844 | RNF103 | ring finger protein 103(RNF103) | Homo sapiens |
| 26994 | RNF11 | ring finger protein 11(RNF11) | Homo sapiens |
| 54778 | RNF111 | ring finger protein 111(RNF111) | Homo sapiens |
| 7732 | RNF112 | ring finger protein 112(RNF112) | Homo sapiens |
| 7737 | RNF113A | ring finger protein 113A(RNF113A) | Homo sapiens |
| 140432 | RNF113B | ring finger protein 113B(RNF113B) | Homo sapiens |
| 55905 | RNF114 | ring finger protein 114(RNF114) | Homo sapiens |
| 27246 | RNF115 | ring finger protein 115(RNF115) | Homo sapiens |
| 55298 | RNF121 | ring finger protein 121(RNF121) | Homo sapiens |
| 79845 | RNF122 | ring finger protein 122(RNF122) | Homo sapiens |
| 63891 | RNF123 | ring finger protein 123(RNF123) | Homo sapiens |
| 54941 | RNF125 | ring finger protein 125(RNF125) | Homo sapiens |
| 55658 | RNF126 | ring finger protein 126(RNF126) | Homo sapiens |
| 79589 | RNF128 | ring finger protein 128, E3 ubiquitin protein ligase(RNF128) | Homo sapiens |
| 11342 | RNF13 | ring finger protein 13(RNF13) | Homo sapiens |
| 55819 | RNF130 | ring finger protein 130(RNF130) | Homo sapiens |
| 168433 | RNF133 | ring finger protein 133(RNF133) | Homo sapiens |
| 84282 | RNF135 | ring finger protein 135(RNF135) | Homo sapiens |
| 51444 | RNF138 | ring finger protein 138(RNF138) | Homo sapiens |
| 11236 | RNF139 | ring finger protein 139(RNF139) | Homo sapiens |
| 9604 | RNF14 | ring finger protein 14(RNF14) | Homo sapiens |
| 50862 | RNF141 | ring finger protein 141(RNF141) | Homo sapiens |
| 9781 | RNF144A | ring finger protein 144A(RNF144A) | Homo sapiens |
| 255488 | RNF144B | ring finger protein 144B(RNF144A) | Homo sapiens |
| 153830 | RNF145 | ring finger protein 145(RNF145) | Homo sapiens |
| 81847 | RNF146 | ring finger protein 146(RNF146) | Homo sapiens |
| 378925 | RNF148 | ring finger protein 148(RNF148) | Homo sapiens |
| 284996 | RNF149 | ring finger protein 149(RNF149) | Homo sapiens |
| 57484 | RNF150 | ring finger protein 150(RNF150) | Homo sapiens |
| 146310 | RNF151 | ring finger protein 151(RNF151) | Homo sapiens |
| 220441 | RNF152 | ring finger protein 152(RNF152) | Homo sapiens |
| 114804 | RNF157 | ring finger protein 157(RNF157) | Homo sapiens |
| 26046 | LTN1 (RNF160) | listerin E3 ubiquitin protein ligase 1(LTN1) | Homo sapiens |
| 494470 | RNF165 | ring finger protein 165(RNF165) | Homo sapiens |
| 115992 | RNF166 | ring finger protein 166(RNF166) | Homo sapiens |
| 26001 | RNF167 | ring finger protein 167(RNF167) | Homo sapiens |
| 165918 | RNF168 | ring finger protein 168(RNF168) | Homo sapiens |
| 254225 | RNF169 | ring finger protein 169(RNF169) | Homo sapiens |
| 56163 | RNF17 | ring finger protein 17(RNF17) | Homo sapiens |
| 81790 | RNF170 | ring finger protein 170(RNF170) | Homo sapiens |
| 285533 | RNF175 | ring finger protein 175(RNF175) | Homo sapiens |
| 285671 | RNF180 | ring finger protein 180(RNF180) | Homo sapiens |
| 51255 | RNF181 | ring finger protein 181(RNF181) | Homo sapiens |
| 221687 | RNF182 | ring finger protein 182(RNF182) | Homo sapiens |
| 138065 | RNF183 | ring finger protein 183(RNF183) | Homo sapiens |
| 91445 | RNF185 | ring finger protein 185(RNF185) | Homo sapiens |
| 54546 | RNF186 | ring finger protein 186(RNF186) | Homo sapiens |
| 149603 | RNF187 | ring finger protein 187(RNF187) | Homo sapiens |
| 25897 | RNF19A | ring finger protein 19A, RBR E3 ubiquitin protein ligase(RNF19A) | Homo sapiens |
| 127544 | RNF19B | ring finger protein 19B(RNF19B) | Homo sapiens |
| 6045 | RNF2 | ring finger protein 2(RNF2) | Homo sapiens |
| 56254 | RNF20 | ring finger protein 20(RNF20) | Homo sapiens |
| 388591 | RNF207 | ring finger protein 207(RNF207) | Homo sapiens |
| 727800 | RNF208 | ring finger protein 208(RNF208) | Homo sapiens |
| 285498 | RNF212 | ring finger protein 212(RNF212) | Homo sapiens |
| 57674 | RNF213 | ring finger protein 213(RNF213) | Homo sapiens |
| 257160 | RNF214 | ring finger protein 214(RNF214) | Homo sapiens |
| 200312 | RNF215 | ring finger protein 215(RNF215) | Homo sapiens |
| 54476 | RNF216 | ring finger protein 216(RNF216) | Homo sapiens |
| 154214 | RNF217 | ring finger protein 217(RNF217) | Homo sapiens |
| 79596 | RNF219 | ring finger protein 219(RNF219) | Homo sapiens |
| 55182 | RNF220 | ring finger protein 220(RNF220) | Homo sapiens |
| 11237 | RNF24 | ring finger protein 24(RNF24) | Homo sapiens |
| 64320 | RNF25 | ring finger protein 25(RNF25) | Homo sapiens |
| 79102 | RNF26 | ring finger protein 26(RNF26) | Homo sapiens |
| 55072 | RNF31 | ring finger protein 31(RNF31) | Homo sapiens |
| 140545 | RNF32 | ring finger protein 32(RNF32) | Homo sapiens |
| 80196 | RNF34 | ring finger protein 34(RNF34) | Homo sapiens |
| 152006 | RNF38 | ring finger protein 38(RNF38) | Homo sapiens |
| 80352 | RNF39 | ring finger protein 39(RNF39) | Homo sapiens |
| 6047 | RNF4 | ring finger protein 4(RNF4) | Homo sapiens |
| 9810 | RNF40 | ring finger protein 40(RNF40) | Homo sapiens |
| 10193 | RNF41 | ring finger protein 41(RNF41) | Homo sapiens |
| 54894 | RNF43 | ring finger protein 43(RNF43) | Homo sapiens |
| 22838 | RNF44 | ring finger protein 44(RNF44) | Homo sapiens |
| 6048 | RNF5 | ring finger protein 5(RNF5) | Homo sapiens |
| 6049 | RNF6 | ring finger protein 6(RNF6) | Homo sapiens |
| 9616 | RNF7 | ring finger protein 7(RNF7) | Homo sapiens |
| 9025 | RNF8 | ring finger protein 8(RNF8) | Homo sapiens |
| 51136 | RNFT1 | ring finger protein, transmembrane 1(RNFT1) | Homo sapiens |
| 89970 | RSPRY1 | ring finger and SPRY domain containing 1(RSPRY1) | Homo sapiens |
| 23450 | SF3B3 | splicing factor 3b subunit 3(SF3B3) | Homo sapiens |
| 57630 | SH3RF1 | SH3 domain containing ring finger 1(SH3RF1) | Homo sapiens |
| 153769 | SH3RF2 | SH3 domain containing ring finger 2(SH3RF2) | Homo sapiens |
| 92799 | SHKBP1 | SH3KBP1 binding protein 1(SHKBP1) | Homo sapiens |
| 257218 | SHPRH | SNF2 histone linker PHD RING helicase(SHPRH) | Homo sapiens |
| 6477 | SIAH1 | siah E3 ubiquitin protein ligase 1(SIAH1) | Homo sapiens |
| 6478 | SIAH2 | siah E3 ubiquitin protein ligase 2(SIAH2) | Homo sapiens |
| 6502 | SKP2 | S-phase kinase associated protein 2(SKP2) | Homo sapiens |
| 57154 | SMURF1 | SMAD specific E3 ubiquitin protein ligase 1(SMURF1) | Homo sapiens |
| 64750 | SMURF2 | SMAD specific E3 ubiquitin protein ligase 2(SMURF2) | Homo sapiens |
| 8651 | SOCS1 | suppressor of cytokine signaling 1(SOCS1) | Homo sapiens |
| 8835 | SOCS2 | suppressor of cytokine signaling 2(SOCS2) | Homo sapiens |
| 9021 | SOCS3 | suppressor of cytokine signaling 3(SOCS3) | Homo sapiens |
| 122809 | SOCS4 | suppressor of cytokine signaling 4(SOCS4) | Homo sapiens |
| 9655 | SOCS5 | suppressor of cytokine signaling 5(SOCS5) | Homo sapiens |
| 9306 | SOCS6 | suppressor of cytokine signaling 6(SOCS6) | Homo sapiens |
| 30837 | SOCS7 | suppressor of cytokine signaling 7(SOCS7) | Homo sapiens |
| 8405 | SPOP | speckle type BTB/POZ protein(SPOP) | Homo sapiens |
| 339745 | SPOPL | speckle type BTB/POZ protein like(SPOPL) | Homo sapiens |
| 84767 | TRIM51 (SPRYD5) | tripartite motif-containing 51(TRIM51) | Homo sapiens |
| 80176 | SPSB1 | splA/ryanodine receptor domain and SOCS box containing 1(SPSB1) | Homo sapiens |
| 84727 | SPSB2 | splA/ryanodine receptor domain and SOCS box containing 2(SPSB2) | Homo sapiens |
| 90864 | SPSB3 | splA/ryanodine receptor domain and SOCS box containing 3(SPSB3) | Homo sapiens |
| 92369 | SPSB4 | splA/ryanodine receptor domain and SOCS box containing 4(SPSB4) | Homo sapiens |
| 10273 | STUB1 | STIP1 homology and U-box containing protein 1(STUB1) | Homo sapiens |
| 84447 | SYVN1 | synoviolin 1(SYVN1) | Homo sapiens |
| 6924 | TCEB3 | transcription elongation factor B subunit 3(TCEB3) | Homo sapiens |
| 7126 | TNFAIP1 | TNF alpha induced protein 1(TNFAIP1) | Homo sapiens |
| 7128 | TNFAIP3 | TNF alpha induced protein 3(TNFAIP3) | Homo sapiens |
| 10210 | TOPORS | TOP1 binding arginine/serine rich protein(TOPORS) | Homo sapiens |
| 7186 | TRAF2 | TNF receptor associated factor 2(TRAF2) | Homo sapiens |
| 7187 | TRAF3 | TNF receptor associated factor 3(TRAF3) | Homo sapiens |
| 9618 | TRAF4 | TNF receptor associated factor 4(TRAF4) | Homo sapiens |
| 7188 | TRAF5 | TNF receptor associated factor 5(TRAF5) | Homo sapiens |
| 7189 | TRAF6 | TNF receptor associated factor 6(TRAF6) | Homo sapiens |
| 84231 | TRAF7 | TNF receptor associated factor 7(TRAF7) | Homo sapiens |
| 10293 | TRAIP | TRAF interacting protein(TRAIP) | Homo sapiens |
| 10107 | TRIM10 | tripartite motif containing 10(TRIM10) | Homo sapiens |
| 81559 | TRIM11 | tripartite motif containing 11(TRIM11) | Homo sapiens |
| 10206 | TRIM13 | tripartite motif containing 13(TRIM13) | Homo sapiens |
| 89870 | TRIM15 | tripartite motif containing 15(TRIM15) | Homo sapiens |
| 51127 | TRIM17 | tripartite motif containing 17(TRIM17) | Homo sapiens |
| 23321 | TRIM2 | tripartite motif containing 2(TRIM2) | Homo sapiens |
| 6737 | TRIM21 | tripartite motif containing 21(TRIM21) | Homo sapiens |
| 10346 | TRIM22 | tripartite motif containing 22(TRIM22) | Homo sapiens |
| 373 | TRIM23 | tripartite motif containing 23(TRIM23) | Homo sapiens |
| 8805 | TRIM24 | tripartite motif containing 24(TRIM24) | Homo sapiens |
| 7706 | TRIM25 | tripartite motif containing 25(TRIM25) | Homo sapiens |
| 7726 | TRIM26 | tripartite motif containing 26(TRIM26) | Homo sapiens |
| 5987 | TRIM27 | tripartite motif containing 27(TRIM27) | Homo sapiens |
| 10155 | TRIM28 | tripartite motif containing 28(TRIM28) | Homo sapiens |
| 10612 | TRIM3 | tripartite motif containing 3(TRIM3) | Homo sapiens |
| 11074 | TRIM31 | tripartite motif containing 31(TRIM31) | Homo sapiens |
| 22954 | TRIM32 | tripartite motif containing 32(TRIM32) | Homo sapiens |
| 51592 | TRIM33 | tripartite motif containing 33(TRIM33) | Homo sapiens |
| 53840 | TRIM34 | tripartite motif containing 34(TRIM34) | Homo sapiens |
| 23087 | TRIM35 | tripartite motif containing 35(TRIM35) | Homo sapiens |
| 55521 | TRIM36 | tripartite motif containing 36(TRIM36) | Homo sapiens |
| 4591 | TRIM37 | tripartite motif containing 37(TRIM37) | Homo sapiens |
| 10475 | TRIM38 | tripartite motif containing 38(TRIM38) | Homo sapiens |
| 56658 | TRIM39 | tripartite motif containing 39(TRIM39) | Homo sapiens |
| 89122 | TRIM4 | tripartite motif containing 4(TRIM4) | Homo sapiens |
| 135644 | TRIM40 | tripartite motif containing 40(TRIM40) | Homo sapiens |
| 90933 | TRIM41 | tripartite motif containing 41(TRIM41) | Homo sapiens |
| 287015 | TRIM42 | tripartite motif containing 42(TRIM42) | Homo sapiens |
| 129868 | TRIM43 | tripartite motif containing 43(TRIM43) | Homo sapiens |
| 80263 | TRIM45 | tripartite motif containing 45(TRIM45) | Homo sapiens |
| 80128 | TRIM46 | tripartite motif containing 46(TRIM46) | Homo sapiens |
| 91107 | TRIM47 | tripartite motif containing 47(TRIM47) | Homo sapiens |
| 79097 | TRIM48 | tripartite motif containing 48(TRIM48) | Homo sapiens |
| 57093 | TRIM49 | tripartite motif containing 49(TRIM49) | Homo sapiens |
| 85363 | TRIM5 | tripartite motif containing 5(TRIM5) | Homo sapiens |
| 135892 | TRIM50 | tripartite motif containing 50(TRIM50) | Homo sapiens |
| 84851 | TRIM52 | tripartite motif containing 52(TRIM52) | Homo sapiens |
| 57159 | TRIM54 | tripartite motif containing 54(TRIM54) | Homo sapiens |
| 84675 | TRIM55 | tripartite motif containing 55(TRIM55) | Homo sapiens |
| 81844 | TRIM56 | tripartite motif containing 56(TRIM56) | Homo sapiens |
| 25893 | TRIM58 | tripartite motif containing 58(TRIM58) | Homo sapiens |
| 286827 | TRIM59 | tripartite motif containing 59(TRIM59) | Homo sapiens |
| 117854 | TRIM6 | tripartite motif containing 6(TRIM6) | Homo sapiens |
| 166655 | TRIM60 | tripartite motif containing 60(TRIM60) | Homo sapiens |
| 391712 | TRIM61 | tripartite motif containing 61(TRIM61) | Homo sapiens |
| 55223 | TRIM62 | tripartite motif containing 62(TRIM62) | Homo sapiens |
| 84676 | TRIM63 | tripartite motif containing 63(TRIM63) | Homo sapiens |
| 120146 | TRIM64 | tripartite motif containing 64(TRIM64) | Homo sapiens |
| 201292 | TRIM65 | tripartite motif containing 65(TRIM65) | Homo sapiens |
| 440730 | TRIM67 | tripartite motif containing 67(TRIM67) | Homo sapiens |
| 55128 | TRIM68 | tripartite motif containing 68(TRIM68) | Homo sapiens |
| 140691 | TRIM69 | tripartite motif containing 69(TRIM69) | Homo sapiens |
| 81786 | TRIM7 | tripartite motif containing 7(TRIM7) | Homo sapiens |
| 493829 | TRIM72 | tripartite motif containing 72(TRIM72) | Homo sapiens |
| 378108 | TRIM74 | tripartite motif containing 74(TRIM74) | Homo sapiens |
| 390231 | TRIM77 | tripartite motif containing 77(TRIM77) | Homo sapiens |
| 81603 | TRIM8 | tripartite motif containing 8(TRIM8) | Homo sapiens |
| 114088 | TRIM9 | tripartite motif containing 9(TRIM9) | Homo sapiens |
| 339976 | TRIML1 | tripartite motif family like 1(TRIML1) | Homo sapiens |
| 9320 | TRIP12 | thyroid hormone receptor interactor 12(TRIP12) | Homo sapiens |
| 7267 | TTC3 | tetratricopeptide repeat domain 3(TTC3) | Homo sapiens |
| 56995 | TULP4 | tubby like protein 4(TULP4) | Homo sapiens |
| 7337 | UBE3A | ubiquitin protein ligase E3A(UBE3A) | Homo sapiens |
| 89910 | UBE3B | ubiquitin protein ligase E3B(UBE3B) | Homo sapiens |
| 9690 | UBE3C | ubiquitin protein ligase E3C(UBE3C) | Homo sapiens |
| 9354 | UBE4A | ubiquitination factor E4A(UBE4A) | Homo sapiens |
| 10277 | UBE4B | ubiquitination factor E4B(UBE4B) | Homo sapiens |
| 22888 | UBOX5 | U-box domain containing 5(UBOX5) | Homo sapiens |
| 22888 | UBOX5 | U-box domain containing 5(UBOX5) | Homo sapiens |
| 197131 | UBR1 | ubiquitin protein ligase E3 component n-recognin 1(UBR1) | Homo sapiens |
| 23304 | UBR2 | ubiquitin protein ligase E3 component n-recognin 2(UBR2) | Homo sapiens |
| 130507 | UBR3 | ubiquitin protein ligase E3 component n-recognin 3 (putative)(UBR3) | Homo sapiens |
| 23352 | UBR4 | ubiquitin protein ligase E3 component n-recognin 4(UBR4) | Homo sapiens |
| 51366 | UBR5 | ubiquitin protein ligase E3 component n-recognin 5(UBR5) | Homo sapiens |
| 29128 | UHRF1 | ubiquitin like with PHD and ring finger domains 1(UHRF1) | Homo sapiens |
| 115426 | UHRF2 | ubiquitin like with PHD and ring finger domains 2(UHRF2) | Homo sapiens |
| 85451 | UNK | unkempt family zinc finger(UNK) | Homo sapiens |
| 64718 | UNKL | unkempt family like zinc finger(UNKL) | Homo sapiens |
| 7428 | VHL | von Hippel-Lindau tumor suppressor(VHL) | Homo sapiens |
| 55823 | VPS11 | VPS11, CORVET/HOPS core subunit(VPS11) | Homo sapiens |
| 57617 | VPS18 | VPS18, CORVET/HOPS core subunit(VPS18) | Homo sapiens |
| 27072 | VPS41 | VPS41, HOPS complex subunit(VPS41) | Homo sapiens |
| 23355 | VPS8 | VPS8, CORVET complex subunit(VPS8) | Homo sapiens |
| 151525 | WDSUB1 | WD repeat, sterile alpha motif and U-box domain containing 1(WDSUB1) | Homo sapiens |
| 7468 | WHSC1 | Wolf-Hirschhorn syndrome candidate 1(WHSC1) | Homo sapiens |
| 26118 | WSB1 | WD repeat and SOCS box containing 1(WSB1) | Homo sapiens |
| 55884 | WSB2 | WD repeat and SOCS box containing 2(WSB2) | Homo sapiens |
| 11059 | WWP1 | WW domain containing E3 ubiquitin protein ligase 1(WWP1) | Homo sapiens |
| 11060 | WWP2 | WW domain containing E3 ubiquitin protein ligase 2(WWP2) | Homo sapiens |
| 331 | XIAP | X-linked inhibitor of apoptosis(XIAP) | Homo sapiens |
| 22890 | ZBTB1 | zinc finger and BTB domain containing 1(ZBTB1) | Homo sapiens |
| 65986 | ZBTB10 | zinc finger and BTB domain containing 10(ZBTB10) | Homo sapiens |
| 27107 | ZBTB11 | zinc finger and BTB domain containing 11(ZBTB11) | Homo sapiens |
| 221527 | ZBTB12 | zinc finger and BTB domain containing 12(ZBTB12) | Homo sapiens |
| 7704 | ZBTB16 | zinc finger and BTB domain containing 16(ZBTB16) | Homo sapiens |
| 7709 | ZBTB17 | zinc finger and BTB domain containing 17(ZBTB17) | Homo sapiens |
| 57621 | ZBTB2 | zinc finger and BTB domain containing 2(ZBTB2) | Homo sapiens |
| 26137 | ZBTB20 | zinc finger and BTB domain containing 20(ZBTB20) | Homo sapiens |
| 9278 | ZBTB22 | zinc finger and BTB domain containing 22(ZBTB22) | Homo sapiens |
| 9841 | ZBTB24 | zinc finger and BTB domain containing 24(ZBTB24) | Homo sapiens |
| 7597 | ZBTB25 | zinc finger and BTB domain containing 25(ZBTB25) | Homo sapiens |
| 57684 | ZBTB26 | zinc finger and BTB domain containing 26(ZBTB26) | Homo sapiens |
| 79842 | ZBTB3 | zinc finger and BTB domain containing 3(ZBTB3) | Homo sapiens |
| 27033 | ZBTB32 | zinc finger and BTB domain containing 32(ZBTB32) | Homo sapiens |
| 10009 | ZBTB33 | zinc finger and BTB domain containing 33(ZBTB33) | Homo sapiens |
| 84614 | ZBTB37 | zinc finger and BTB domain containing 37(ZBTB37) | Homo sapiens |
| 253461 | ZBTB38 | zinc finger and BTB domain containing 38(ZBTB38) | Homo sapiens |
| 9880 | ZBTB39 | zinc finger and BTB domain containing 39(ZBTB39) | Homo sapiens |
| 57659 | ZBTB4 | zinc finger and BTB domain containing 4(ZBTB4) | Homo sapiens |
| 9923 | ZBTB40 | zinc finger and BTB domain containing 40(ZBTB40) | Homo sapiens |
| 360023 | ZBTB41 | zinc finger and BTB domain containing 41(ZBTB41) | Homo sapiens |
| 23099 | ZBTB43 | zinc finger and BTB domain containing 43(ZBTB43) | Homo sapiens |
| 29068 | ZBTB44 | zinc finger and BTB domain containing 44(ZBTB44) | Homo sapiens |
| 84878 | ZBTB45 | zinc finger and BTB domain containing 45(ZBTB45) | Homo sapiens |
| 140685 | ZBTB46 | zinc finger and BTB domain containing 46(ZBTB46) | Homo sapiens |
| 3104 | ZBTB48 | zinc finger and BTB domain containing 48(ZBTB48) | Homo sapiens |
| 166793 | ZBTB49 | zinc finger and BTB domain containing 49(ZBTB49) | Homo sapiens |
| 9925 | ZBTB5 | zinc finger and BTB domain containing 5(ZBTB5) | Homo sapiens |
| 10773 | ZBTB6 | zinc finger and BTB domain containing 6(ZBTB6) | Homo sapiens |
| 51341 | ZBTB7A | zinc finger and BTB domain containing 7A(ZBTB7A) | Homo sapiens |
| 51043 | ZBTB7B | zinc finger and BTB domain containing 7B(ZBTB7B) | Homo sapiens |
| 201501 | ZBTB7C | zinc finger and BTB domain containing 7C(ZBTB7C) | Homo sapiens |
| 653121 | ZBTB8A | zinc finger and BTB domain containing 8A(ZBTB8A) | Homo sapiens |
| 221504 | ZBTB9 | zinc finger and BTB domain containing 9(ZBTB9) | Homo sapiens |
| 60685 | ZFAND3 | zinc finger AN1-type containing 3(ZFAND3) | Homo sapiens |
| 7763 | ZFAND5 | zinc finger AN1-type containing 5(ZFAND5) | Homo sapiens |
| 54469 | ZFAND6 | zinc finger AN1-type containing 6(ZFAND6) | Homo sapiens |
| 7541 | ZBTB14 (ZFP161) | zinc finger and BTB domain containing 14(ZBTB14) | Homo sapiens |
| 7542 | ZFPL1 | zinc finger protein like 1(ZFPL1) | Homo sapiens |
| 7690 | ZNF131 | zinc finger protein 131(ZNF131) | Homo sapiens |
| 10472 | ZBTB18 (ZNF238) | zinc finger and BTB domain containing 18(ZBTB18) | Homo sapiens |
| 49854 | ZBTB21 (ZNF295) | zinc finger and BTB domain containing 21(ZBTB21) | Homo sapiens |
| 90850 | ZNF598 | zinc finger protein 598(ZNF598) | Homo sapiens |
| 158506 | ZNF645 | zinc finger protein 645(ZNF645) | Homo sapiens |
| 84937 | ZNRF1 | zinc and ring finger 1(ZNRF1) | Homo sapiens |
| 223082 | ZNRF2 | zinc and ring finger 2(ZNRF2) | Homo sapiens |
| 84133 | ZNRF3 | zinc and ring finger 3(ZNRF3) | Homo sapiens |
| 148066 | ZNRF4 | zinc and ring finger 4(ZNRF4) | Homo sapiens |
| 151112 | ZSWIM2 | zinc finger SWIM-type containing 2(ZSWIM2) | Homo sapiens |
| PCs |  |  |  |
| 5682 | PSMA1 | proteasome subunit alpha 1(PSMA1) | Homo sapiens |
| 5683 | PSMA2 | proteasome subunit alpha 2(PSMA2) | Homo sapiens |
| 5684 | PSMA3 | proteasome subunit alpha 3(PSMA3) | Homo sapiens |
| 5685 | PSMA4 | proteasome subunit alpha 4(PSMA4) | Homo sapiens |
| 5686 | PSMA5 | proteasome subunit alpha 5(PSMA5) | Homo sapiens |
| 5687 | PSMA6 | proteasome subunit alpha 6(PSMA6) | Homo sapiens |
| 5688 | PSMA7 | proteasome subunit alpha 7(PSMA7) | Homo sapiens |
| 143471 | PSMA8 | proteasome subunit alpha 8(PSMA8) | Homo sapiens |
| 5689 | PSMB1 | proteasome subunit beta 1(PSMB1) | Homo sapiens |
| 5699 | PSMB10 | proteasome subunit beta 10(PSMB10) | Homo sapiens |
| 122706 | PSMB11 | proteasome subunit beta 11(PSMB11) | Homo sapiens |
| 5690 | PSMB2 | proteasome subunit beta 2(PSMB2) | Homo sapiens |
| 5691 | PSMB3 | proteasome subunit beta 3(PSMB3) | Homo sapiens |
| 5692 | PSMB4 | proteasome subunit beta 4(PSMB4) | Homo sapiens |
| 5693 | PSMB5 | proteasome subunit beta 5(PSMB5) | Homo sapiens |
| 5694 | PSMB6 | proteasome subunit beta 6(PSMB6) | Homo sapiens |
| 5695 | PSMB7 | proteasome subunit beta 7(PSMB7) | Homo sapiens |
| 5696 | PSMB8 | proteasome subunit beta 8(PSMB8) | Homo sapiens |
| 5698 | PSMB9 | proteasome subunit beta 9(PSMB9) | Homo sapiens |
| 5700 | PSMC1 | proteasome 26S subunit, ATPase 1(PSMC1) | Homo sapiens |
| 5701 | PSMC2 | proteasome 26S subunit, ATPase 2(PSMC2) | Homo sapiens |
| 5702 | PSMC3 | proteasome 26S subunit, ATPase 3(PSMC3) | Homo sapiens |
| 29893 | PSMC3IP | PSMC3 interacting protein(PSMC3IP) | Homo sapiens |
| 5704 | PSMC4 | proteasome 26S subunit, ATPase 4(PSMC4) | Homo sapiens |
| 5705 | PSMC5 | proteasome 26S subunit, ATPase 5(PSMC5) | Homo sapiens |
| 5706 | PSMC6 | proteasome 26S subunit, ATPase 6(PSMC6) | Homo sapiens |
| 5707 | PSMD1 | proteasome 26S subunit, non-ATPase 1(PSMD1) | Homo sapiens |
| 5716 | PSMD10 | proteasome 26S subunit, non-ATPase 10(PSMD10) | Homo sapiens |
| 5717 | PSMD11 | proteasome 26S subunit, non-ATPase 11(PSMD11) | Homo sapiens |
| 5718 | PSMD12 | proteasome 26S subunit, non-ATPase 12(PSMD12) | Homo sapiens |
| 5719 | PSMD13 | proteasome 26S subunit, non-ATPase 13(PSMD13) | Homo sapiens |
| 10213 | PSMD14 | proteasome 26S subunit, non-ATPase 14(PSMD14) | Homo sapiens |
| 5708 | PSMD2 | proteasome 26S subunit, non-ATPase 2(PSMD2) | Homo sapiens |
| 5709 | PSMD3 | proteasome 26S subunit, non-ATPase 3(PSMD3) | Homo sapiens |
| 5710 | PSMD4 | proteasome 26S subunit, non-ATPase 4(PSMD4) | Homo sapiens |
| 5711 | PSMD5 | proteasome 26S subunit, non-ATPase 5(PSMD5) | Homo sapiens |
| 9861 | PSMD6 | proteasome 26S subunit, non-ATPase 6(PSMD6) | Homo sapiens |
| 5713 | PSMD7 | proteasome 26S subunit, non-ATPase 7(PSMD7) | Homo sapiens |
| 5714 | PSMD8 | proteasome 26S subunit, non-ATPase 8(PSMD8) | Homo sapiens |
| 5715 | PSMD9 | proteasome 26S subunit, non-ATPase 9(PSMD9) | Homo sapiens |
| 5720 | PSME1 | proteasome activator subunit 1(PSME1) | Homo sapiens |
| 5721 | PSME2 | proteasome activator subunit 2(PSME2) | Homo sapiens |
| 10197 | PSME3 | proteasome activator subunit 3(PSME3) | Homo sapiens |
| 23198 | PSME4 | proteasome activator subunit 4(PSME4) | Homo sapiens |
| 9491 | PSMF1 | proteasome inhibitor subunit 1(PSMF1) | Homo sapiens |
| 8624 | PSMG1 | proteasome assembly chaperone 1(PSMG1) | Homo sapiens |
| 56984 | PSMG2 | proteasome assembly chaperone 2(PSMG2) | Homo sapiens |
| 84262 | PSMG3 | proteasome assembly chaperone 3(PSMG3) | Homo sapiens |
| 389362 | PSMG4 | proteasome assembly chaperone 4(PSMG4) | Homo sapiens |
| 51371 | POMP | proteasome maturation protein(POMP) | Homo sapiens |
| 23392 | KIAA0368 | KIAA0368(KIAA0368) | Homo sapiens |
| 11047 | ADRM1 | adhesion regulating molecule 1(ADRM1) | Homo sapiens |
| 80227 | PAAF1 | proteasomal ATPase associated factor 1（PAAF1） | Homo sapiens |
| DUBs |  |  |  |
| 4287 | ATXN3 | ataxin 3(ATXN3) | Homo sapiens |
| 92552 | ATXN3L | ataxin 3 like(ATXN3L) | Homo sapiens |
| 8314 | BAP1 | BRCA1 associated protein 1(BAP1) | Homo sapiens |
| 79184 | BRCC3 | BRCA1/BRCA2-containing complex subunit 3(BRCC3) | Homo sapiens |
| 10987 | COPS5 | COP9 signalosome subunit 5(COPS5) | Homo sapiens |
| 10980 | COPS6 | COP9 signalosome subunit 6(COPS6) | Homo sapiens |
| 1540 | CYLD | CYLD lysine 63 deubiquitinase(CYLD) | Homo sapiens |
| 8665 | EIF3F | eukaryotic translation initiation factor 3 subunit F(EIF3F) | Homo sapiens |
| 8667 | EIF3H | eukaryotic translation initiation factor 3 subunit H(EIF3H) | Homo sapiens |
| 9929 | JOSD1 | Josephin domain containing 1(JOSD1) | Homo sapiens |
| 126119 | JOSD2 | Josephin domain containing 2(JOSD2) | Homo sapiens |
| 84954 | MPND | MPN domain containing(MPND) | Homo sapiens |
| 114803 | MYSM1 | Myb like, SWIRM and MPN domains 1(MYSM1) | Homo sapiens |
| 55611 | OTUB1 | OTU deubiquitinase, ubiquitin aldehyde binding 1(OTUB1) | Homo sapiens |
| 78990 | OTUB2 | OTU deubiquitinase, ubiquitin aldehyde binding 2(OTUB2) | Homo sapiens |
| 220213 | OTUD1 | OTU deubiquitinase 1(OTUD1) | Homo sapiens |
| 23252 | OTUD3 | OTU deubiquitinase 3(OTUD3) | Homo sapiens |
| 54726 | OTUD4 | OTU deubiquitinase 4(OTUD4) | Homo sapiens |
| 55593 | OTUD5 | OTU deubiquitinase 5(OTUD5) | Homo sapiens |
| 139562 | OTUD6A | OTU deubiquitinase 6A(OTUD6A) | Homo sapiens |
| 51633 | OTUD6B | OTU domain containing 6B(OTUD6B) | Homo sapiens |
| 161725 | OTUD7A | OTU deubiquitinase 7A(OTUD7A) | Homo sapiens |
| 56957 | OTUD7B | OTU deubiquitinase 7B(OTUD7B) | Homo sapiens |
| 9924 | PAN2 | PAN2 poly(A) specific ribonuclease subunit | Homo sapiens |
| 10594 | PRPF8 | pre-mRNA processing factor 8(PRPF8) | Homo sapiens |
| 10213 | PSMD14 | proteasome 26S subunit, non-ATPase 14(PSMD14) | Homo sapiens |
| 5713 | PSMD7 | proteasome 26S subunit, non-ATPase 7(PSMD7) | Homo sapiens |
| 10617 | STAMBP | STAM binding protein(STAMBP) | Homo sapiens |
| 57559 | STAMBPL1 | STAM binding protein like 1(STAMBPL1) | Homo sapiens |
| 7128 | TNFAIP3 | TNF alpha induced protein 3(TNFAIP3) | Homo sapiens |
| 7345 | UCHL1 | ubiquitin C-terminal hydrolase L1(UCHL1) | Homo sapiens |
| 7347 | UCHL3 | ubiquitin C-terminal hydrolase L3(UCHL3) | Homo sapiens |
| 51377 | UCHL5 | ubiquitin C-terminal hydrolase L5(UCHL5) | Homo sapiens |
| 7398 | USP1 | ubiquitin specific peptidase 1(USP1) | Homo sapiens |
| 9100 | USP10 | ubiquitin specific peptidase 10(USP10) | Homo sapiens |
| 8237 | USP11 | ubiquitin specific peptidase 11(USP11) | Homo sapiens |
| 219333 | USP12 | ubiquitin specific peptidase 12(USP12) | Homo sapiens |
| 8975 | USP13 | ubiquitin specific peptidase 13 (isopeptidase T-3)(USP13) | Homo sapiens |
| 9097 | USP14 | ubiquitin specific peptidase 14(USP14) | Homo sapiens |
| 9958 | USP15 | ubiquitin specific peptidase 15(USP15) | Homo sapiens |
| 10600 | USP16 | ubiquitin specific peptidase 16(USP16) | Homo sapiens |
| 391627 | USP17 | ubiquitin specific peptidase 17-like family member 9, pseudogene (USP17L9P) | Homo sapiens |
| 377630 | USP17L2 | ubiquitin specific peptidase 17-like family member 2(USP17L2) | Homo sapiens |
| 391622 | USP17L6P | ubiquitin specific peptidase 17-like family member 6, pseudogene(USP17L6P) | Homo sapiens |
| 11274 | USP18 | ubiquitin specific peptidase 18(USP18) | Homo sapiens |
| 10869 | USP19 | ubiquitin specific peptidase 19(USP19) | Homo sapiens |
| 9099 | USP2 | ubiquitin specific peptidase 2(USP2) | Homo sapiens |
| 10868 | USP20 | ubiquitin specific peptidase 20(USP20) | Homo sapiens |
| 27005 | USP21 | ubiquitin specific peptidase 21(USP21) | Homo sapiens |
| 23326 | USP22 | ubiquitin specific peptidase 22(USP22) | Homo sapiens |
| 23358 | USP24 | ubiquitin specific peptidase 24(USP24) | Homo sapiens |
| 29761 | USP25 | ubiquitin specific peptidase 25(USP25) | Homo sapiens |
| 83844 | USP26 | ubiquitin specific peptidase 26(USP26) | Homo sapiens |
| 389856 | USP27X | ubiquitin specific peptidase 27, X-linked(USP27X) | Homo sapiens |
| 57646 | USP28 | ubiquitin specific peptidase 28(USP28) | Homo sapiens |
| 57663 | USP29 | ubiquitin specific peptidase 29(USP29) | Homo sapiens |
| 9960 | USP3 | ubiquitin specific peptidase 3(USP3) | Homo sapiens |
| 84749 | USP30 | ubiquitin specific peptidase 30(USP30) | Homo sapiens |
| 57478 | USP31 | ubiquitin specific peptidase 31(USP31) | Homo sapiens |
| 84669 | USP32 | ubiquitin specific peptidase 32(USP32) | Homo sapiens |
| 23032 | USP33 | ubiquitin specific peptidase 33(USP33) | Homo sapiens |
| 9736 | USP34 | ubiquitin specific peptidase 34(USP34) | Homo sapiens |
| 57558 | USP35 | ubiquitin specific peptidase 35(USP35) | Homo sapiens |
| 57602 | USP36 | ubiquitin specific peptidase 36(USP36) | Homo sapiens |
| 57695 | USP37 | ubiquitin specific peptidase 37(USP37) | Homo sapiens |
| 84640 | USP38 | ubiquitin specific peptidase 38(USP38) | Homo sapiens |
| 10713 | USP39 | ubiquitin specific peptidase 39(USP39) | Homo sapiens |
| 7375 | USP4 | ubiquitin specific peptidase 4(USP4) | Homo sapiens |
| 55230 | USP40 | ubiquitin specific peptidase 40(USP40) | Homo sapiens |
| 84132 | USP42 | ubiquitin specific peptidase 42(USP42) | Homo sapiens |
| 124739 | USP43 | ubiquitin specific peptidase 43(USP43) | Homo sapiens |
| 84101 | USP44 | ubiquitin specific peptidase 44(USP44) | Homo sapiens |
| 85015 | USP45 | ubiquitin specific peptidase 45(USP45) | Homo sapiens |
| 64854 | USP46 | ubiquitin specific peptidase 46(USP46) | Homo sapiens |
| 55031 | USP47 | ubiquitin specific peptidase 47(USP47) | Homo sapiens |
| 84196 | USP48 | ubiquitin specific peptidase 48(USP48) | Homo sapiens |
| 25862 | USP49 | ubiquitin specific peptidase 49(USP49) | Homo sapiens |
| 8078 | USP5 | ubiquitin specific peptidase 5(USP5) | Homo sapiens |
| 373509 | USP50 | ubiquitin specific peptidase 50(USP50) | Homo sapiens |
| 158880 | USP51 | ubiquitin specific peptidase 51(USP51) | Homo sapiens |
| 54532 | USP53 | ubiquitin specific peptidase 53(USP53) | Homo sapiens |
| 159195 | USP54 | ubiquitin specific peptidase 54(USP54) | Homo sapiens |
| 9098 | USP6 | ubiquitin specific peptidase 6(USP6) | Homo sapiens |
| 9712 | USP6NL | USP6 N-terminal like(USP6NL) | Homo sapiens |
| 7874 | USP7 | ubiquitin specific peptidase 7(USP7) | Homo sapiens |
| 9101 | USP8 | ubiquitin specific peptidase 8(USP8) | Homo sapiens |
| 8239 | USP9X | ubiquitin specific peptidase 9, X-linked(USP9X) | Homo sapiens |
| 8287 | USP9Y | ubiquitin specific peptidase 9, Y-linked(USP9Y) | Homo sapiens |
| 10208 | USPL1 | ubiquitin specific peptidase like 1(USPL1) | Homo sapiens |
| 80124 | VCPIP1 | valosin containing protein interacting protein 1(VCPIP1) | Homo sapiens |
| 55432 | YOD1 | YOD1 deubiquitinase(YOD1) | Homo sapiens |
| 54764 | ZRANB1 | zinc finger RANBP2-type containing 1 (ZRANB1) | Homo sapiens |
