# Supplementary figures and images for: A Ubiquitin-Proteasome Gene Signature for Predicting Prognosis in Patients With Lung Adenocarcinoma
Source: Front Genet. 2022 May 31;13:893511. doi: 10.3389/fgene.2022.893511 (PMC9194557; doi:10.3389/fgene.2022.893511)

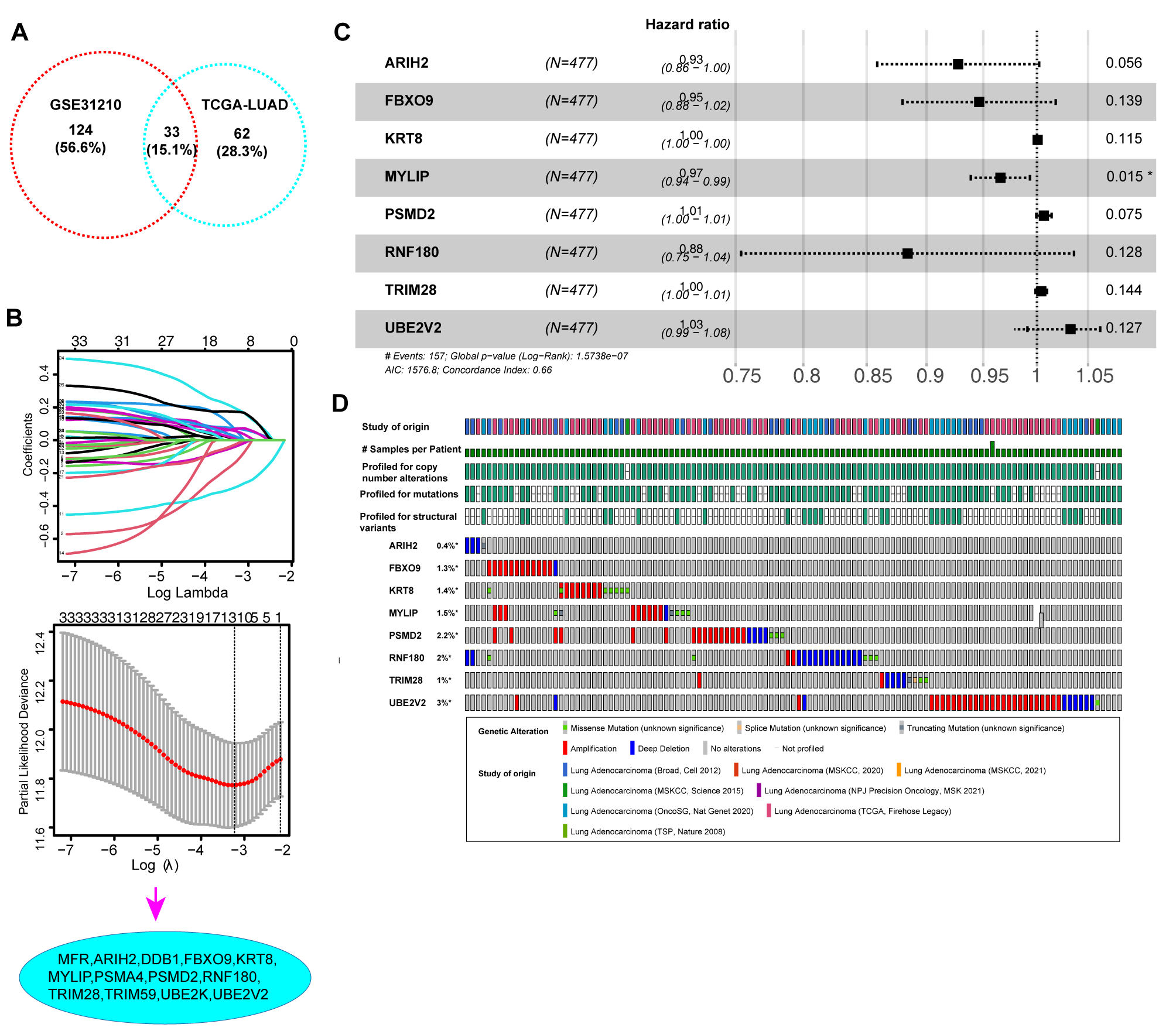

Supplement: Supplementary file 2 [file Image1.tif]
