# Supplementary material for: A Ubiquitin-Proteasome Gene Signature for Predicting Prognosis in Patients With Lung Adenocarcinoma
Source: Front Genet. 2022 May 31;13:893511. doi: 10.3389/fgene.2022.893511 (PMC9194557; doi:10.3389/fgene.2022.893511)
Supplement: Supplementary file 3 [file Image1.PDF]

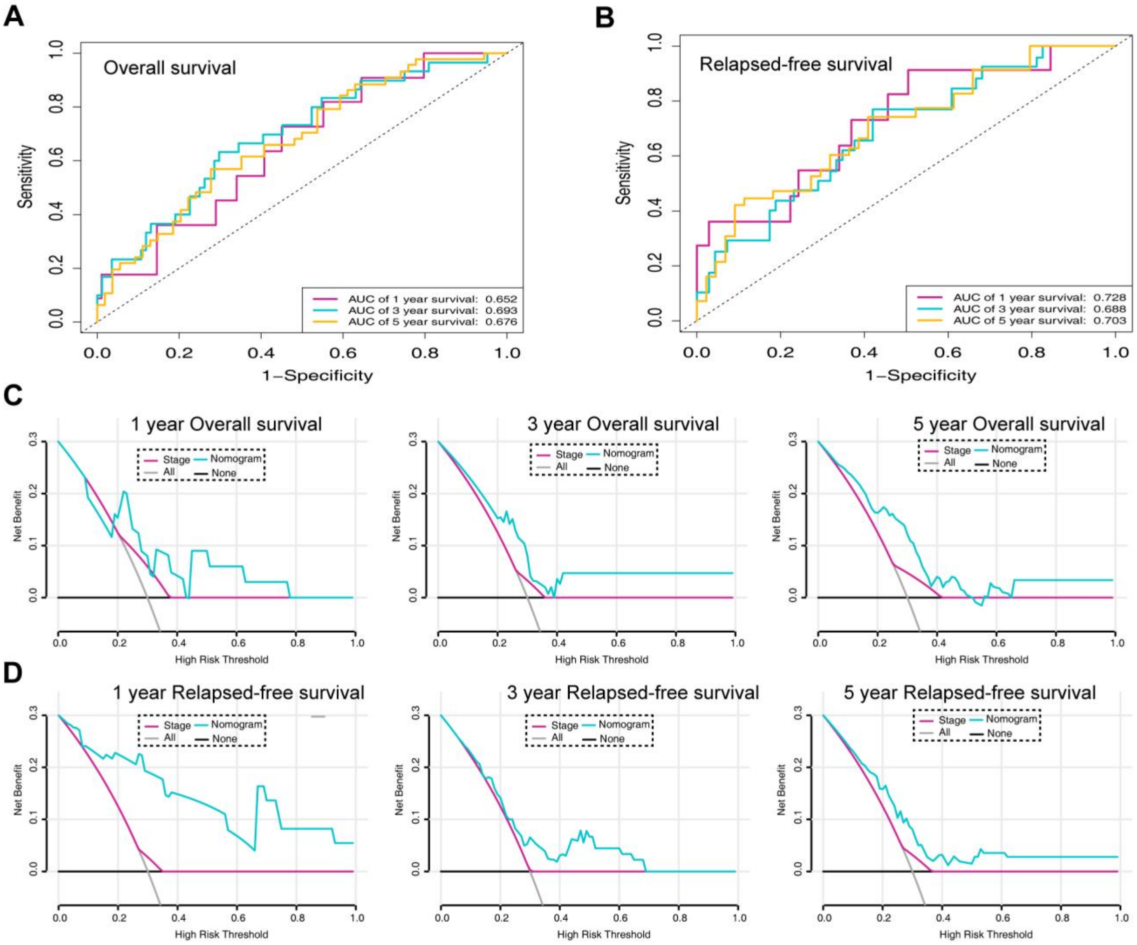

Figure S1: Validated LUAD survival prediction nomogram based on the GSE50081 validation set. (A) Time-dependent receiver operating characteristic curves for nomogram prediction of 1-, 3-, and 5-year overall survival rates. (B) Time-dependent receiver operating characteristic curves for nomogram mediated prediction of 1-, 3-, and 5-year relapse-free survival in LUAD patients. (C) Decision curve analysis for overall survival. (D) Decision curve analysis for relapse-free survival.
